# Development of an algorithm for evaluating the impact of measurement variability on response categorization in oncology trials

**DOI:** 10.1186/s12874-019-0727-7

**Published:** 2019-05-02

**Authors:** Jeong-Hwa Yoon, Soon Ho Yoon, Seokyung Hahn

**Affiliations:** 1 Interdisciplinary Program in Medical Informatics, Seoul National University College of Medicine, Seoul, South Korea; 2 Department of Radiology, Seoul National University College of Medicine, Seoul, South Korea; 3 Medical Statistics Laboratory, Department of Medicine, Seoul National University College of Medicine, 103 Daehak-ro, Jongno-gu, Seoul, 03080 South Korea

**Keywords:** RECIST, Measurement variability, Hierarchical model, Algorithm

## Abstract

**Background:**

Radiologic assessments of baseline and post-treatment tumor burden are subject to measurement variability, but the impact of this variability on the objective response rate (ORR) and progression rate in specific trials has been unpredictable on a practical level. In this study, we aimed to develop an algorithm for evaluating the quantitative impact of measurement variability on the ORR and progression rate.

**Methods:**

First, we devised a hierarchical model for estimating the distribution of measurement variability using a clinical trial dataset of computed tomography scans. Next, a simulation method was used to calculate the probability representing the effect of measurement errors on categorical diagnoses in various scenarios using the estimated distribution. Based on the probabilities derived from the simulation, we developed an algorithm to evaluate the reliability of an ORR (or progression rate) (i.e., the variation in the assessed rate) by generating a 95% central range of ORR (or progression rate) results if a reassessment was performed. Finally, we performed validation using an external dataset. In the validation of the estimated distribution of measurement variability, the coverage level was calculated as the proportion of the 95% central ranges of hypothetical second readings that covered the actual burden sizes. In the validation of the evaluation algorithm, for 100 resampled datasets, the coverage level was calculated as the proportion of the 95% central ranges of ORR results that covered the ORR from a real second assessment.

**Results:**

We built a web tool for implementing the algorithm (publicly available at http://studyanalysis2017.pythonanywhere.com/). In the validation of the estimated distribution and the algorithm, the coverage levels were 93 and 100%, respectively.

**Conclusions:**

The validation exercise using an external dataset demonstrated the adequacy of the statistical model and the utility of the developed algorithm. Quantification of variation in the ORR and progression rate due to potential measurement variability is essential and will help inform decisions made on the basis of trial data.

**Electronic supplementary material:**

The online version of this article (10.1186/s12874-019-0727-7) contains supplementary material, which is available to authorized users.

## Background

In medicine, some diagnoses of diseases or determinations of treatment response are dichotomized or categorized according to changes in continuous data as measured before and after intervention. In oncology, treatment response after anti-cancer therapy is determined based on the percent change of the tumor burden before and after the treatment, as measured by physicians on computed tomography (CT) scans. The tumor burden in a patient is represented by measurements of representative lesions (so-called target lesions), followed by the sum of the measurements. The objective response rate (ORR) and progression rate, which are used in oncology trials, are imaging-based measures of outcomes assessed using the Response Evaluation Criteria in Solid Tumors (RECIST) response categorization according to the percent change of the tumor burden before and after treatment [1]. The categories include complete response, partial response, stable disease, and progression. The ORR and progression rate are defined as the percentage of patients who achieve complete or partial response and progression, respectively [2].

Radiologic measurements of the tumor burden can be inconsistent across assessments, which may affect the assessed percent change of the tumor burden, the response categorization, and the resulting ORR and progression rate [3]. Indeed, it has been suggested that reimaging the same single-tumor burden can result in an increase or decrease of the burden size by less than 10% relative to the first measurement [4], and that when different readers assess the same single-tumor burden before and after treatment, the absolute difference in the values of percent change (%) between readers can be as much as 30% [5]. This measurement variability eventually results in variability in the response classification and ORR [6].

Many oncology trials designate the objective response as a primary or a secondary outcome and provide the ORR as a measure of the outcome, but the reliability of RECIST-based response determinations and the ORR due to measurement variability in oncology trials remains poorly understood [5]. In this study, we developed an algorithm for quantitative evaluation of the impact of measurement variability on the ORR and progression rate, which is an area that has not yet been considered in previous research.

The RECIST-based response could be considered as an ordinal measure. Some statistical methods for assessing the agreement of such ordinal data have been suggested in the literature [7–10]. The response categorization is an ordinal dichotomization of change in tumor burden. Following the RECIST guideline, the tumor burden is essentially calculated by summing the measurements of the various target lesions that can adequately represent the overall tumor burden in a patient. Since measurement error fundamentally occurs when observing lesion sizes, and manifests as measurement variability of tumor burden size, percent change, and response categorization, it is important to understand the primary behavior of measurement variability by modeling measurements of lesion size. The response categorization of tumor burdens of various sizes initially determined to have shown the same response can in fact be differently reproducible based on how or whether the measured size of the tumor burdens is close to the cutoff values for categorization. Such differences should also be taken into account for measuring the uncertainty and reproducibility of the eventual RECIST-based response.

Bland and Altman (1986 and 1999) originally proposed the limits-of-agreement (LOA) method for assessing agreement between measurements by two observers [11, 12]. LOAs provide a straightforward way of evaluating measurement agreement by plotting the measurement difference against the mean of the two measurements. Sometimes the differences between the measurements are dependent on the size of the measurements, with increasing measurement error accompanying an increasing scale of the measurement values, in which case conventional LOAs may not represent the data well. When a distribution of errors is skewed, data are often log-transformed to approximate normality. Euser et al. (2008) discussed a modeling approach to calculate meaningful LOAs on log-transformed data [13].

To develop an algorithm for evaluating the impact of measurement variability on response categorization in oncology, we initially constructed a dataset based on repeated evaluations before and after treatment of the selected lesions in order to enable estimation of the within-lesion component of the measurement variability. We devised an appropriate transformation of the measurements of tumor size using the dataset, with an exploration of LOAs. We constructed a bivariate hierarchical linear mixed-effects model to estimate the distributions of measurement errors before and after treatment, using transformed data to account for the measurement size-dependency of the measurement errors. In the simulation step, the estimated distribution was used to generate a hypothetical second measurement of the original measurement. We then calculated the probability that the second measurement would be designated as a certain response. Using these probability values, we built up an algorithm for evaluating the impact of measurement variability on the ORR or progression rate at the trial level, and facilitated the implementation of this algorithm by developing a web tool. Finally, we performed validations to determine whether the hypothetical second measurements adequately predicted the actual repeatedly read measurements and whether the resulting ORR replicated the ORR as assessed by actual third parties.

This article is organized as follows. The Methods section first introduces the data structure and describes the process of the development of the evaluation algorithm by modeling and simulations, followed by the method of validation. The Results section explains the routines of the developed web tool for implementing the algorithm and interprets the results of the validations. The final section contains a discussion of the usefulness and limitations of our method and the usability and potential of the algorithm.

## Methods

### RECIST guideline

The RECIST guideline is a set of standardized criteria for assessing tumor response after anti-cancer treatment in oncology trials. The guideline mainly deals with how to define a tumor burden, how to measure changes of tumor burden, and how to categorize the response of the tumor after treatment. Following the guideline, the tumor burden is calculated by summing the measurements of the various target lesions that can adequately represent the overall tumor burden in a patient. The percent change is then calculated as a change in the tumor burden after treatment relative to its baseline value as a percentage. The RECIST-based response is finally categorized based on the percent change of the tumor burden. The categories include complete response (− 100%), partial response (− 30% or less), stable disease (− 30 to 20%), and progression (20% or more).

### Data

In order to estimate the distribution of measurement errors through modeling and to develop an algorithm to evaluate the impact of measurement variability on response categorization in oncology, we constructed a dataset by obtaining repeated evaluations of the selected lesions before and after treatment, thereby enabling estimation of the within-lesion component of the measurement variability.

De-identified chest CT scans were initially obtained from 75 patients who were enrolled in an existing phase III randomized trial of chemotherapy for advanced small-cell lung cancer (SCLC). A single radiologist chose a total of 249 measurable target lesions in the 75 patients, and the median number of measurable target lesions per patient was 3. Each target lesion was read by six radiologists in four separate sessions, twice at baseline and twice after treatment.

Twenty-four measurements of the longest diameter (12 at baseline and 12 at follow-up) were taken from each lesion. Of all the target lesions, 119 were on lymph nodes. A short-axis diameter, perpendicular to the longest diameter, was additionally taken from those target lymph nodes. Twenty-four measurements of the shortest diameter (12 at baseline and 12 at follow-up) were taken from each lymph node.

For validation of the estimated distributions of measurement error and the evaluation algorithm, we used another dataset of 56 target lesions from 22 patients with refractory SCLC from a phase II trial. Each target lesion was measured by a within-trial local reader and one external reader in two separate sessions, once at baseline and once after treatment. The longest diameters and short-axis diameters were taken from the solid lesions and lymph nodes, respectively.

### Modeling

**Fig. 1:**
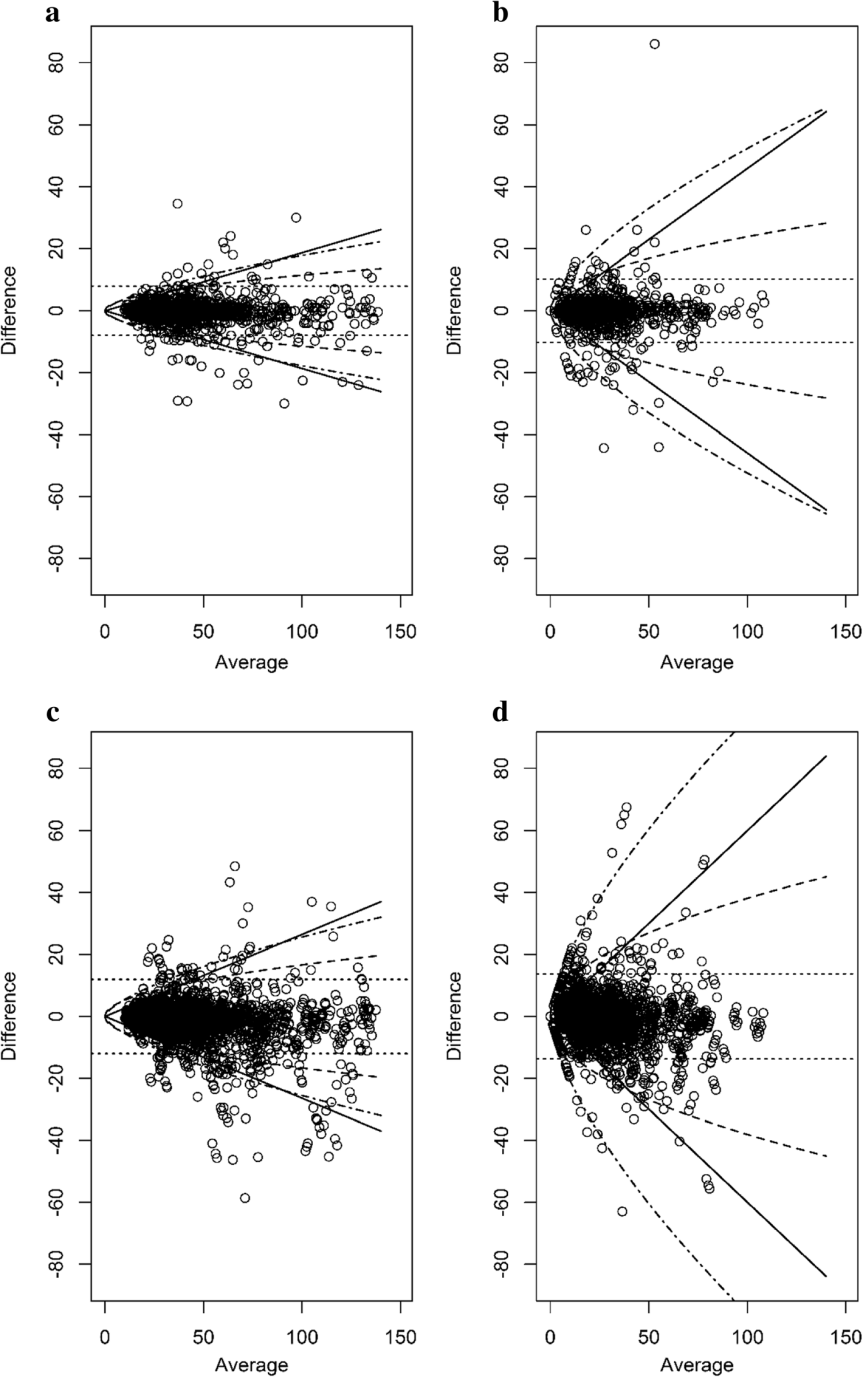
Bland-Altman plot and limits of agreement for the transformation candidates. **a** Intra-reader plot at baseline. The differences between the first and second measurements in relation to the average of the two measurements made by one reader of the tumor burden. **b** Intra-reader plot at post-treatment. The differences between the first and second measurements in relation to the average of the two measurements made by one reader of the tumor burden. **c** Inter-reader plot at baseline. The differences between the measurements in relation to the average of the measurements made by two readers of the tumor burden. **d** Inter-reader plot at post-treatment. The differences between the measurements in relation to the average of the measurements made by two readers of the tumor burden. Dotted lines: limits of agreement (LOA) from the model using the original lesion size; solid lines: LOA from the model using the log-transformation of the lesion size; dot-dashed lines: LOA from the model using cube root-transformation of the lesion size; dashed lines: LOA from the model using square root-transformation of the lesion size

A hierarchical linear mixed-effects model including lesions, readers, and the interaction effects of lesions and readers as random effects was considered based on the measurements obtained from the six reviewers at each of the baseline and the post-treatment phases [14]. We assessed the plausibility of using the square root, cube root, and log transformation of the lesion size as possible assumptions for the distribution of measurement errors. LOA presentations with a Bland-Altman plot [11] were explored at each phase (Fig. 1) (see formulas of the LOAs on the original scale for the *n*th root-transformed variables in Additional file 1). Since the LOA from the square root transformation of the lesion size described the data pattern appropriately, we chose the square root transformation, considering the nonlinear lesion size-dependency of the measurement error. We trimmed off 5% of the outlying data with a large standardized residual based on the considered model using the transformation at each phase [15].

The final model was then fitted in a bivariate form, accounting for a possible correlation between the measurements in the baseline and the post-treatment phases:

$$ \left(\begin{array}{c}\sqrt{Y_{ijk:b}}\\ {}\sqrt{Y_{ijk:p}}\end{array}\right) $$= $$ \left(\begin{array}{c}{\mu}_b\\ {}{\mu}_p\end{array}\right) $$+ $$ \left(\begin{array}{c}{\alpha}_{i:b}\\ {}{\alpha}_{i:p}\end{array}\right) $$ + $$ \left(\begin{array}{c}{\beta}_{j:b}\\ {}{\beta}_{j:p}\end{array}\right) $$ + $$ \left(\begin{array}{c}{\gamma}_{ij:b}\\ {}{\gamma}_{ij:p}\end{array}\right) $$ + $$ \left(\begin{array}{c}{\varepsilon}_{(ij)k:b}\\ {}{\varepsilon}_{(ij)k:p}\end{array}\right) $$, where $$ \left(\begin{array}{c}\sqrt{Y_{ijk:b}}\\ {}\sqrt{Y_{ijk:p}}\end{array}\right) $$ denotes the square root-transformed measures of the lesion diameters at baseline and post-treatment (*b* : baseline,  *p* : post − treatment) and $$ \left(\begin{array}{c}{\mu}_b\\ {}{\mu}_p\end{array}\right) $$ are the fixed parameters. In addition, $$ \left(\begin{array}{c}{\alpha}_{i:b}\\ {}{\alpha}_{i:p}\end{array}\right) $$, the lesion effect, $$ \left(\begin{array}{c}{\beta}_{j:b}\\ {}{\beta}_{j:p}\end{array}\right) $$, the reader effect, and $$ \left(\begin{array}{c}{\gamma}_{ij:b}\\ {}{\gamma}_{ij:p}\end{array}\right) $$, the interaction between the lesion and reader, are random effects normally distributed with a mean vector of $$ \left(\begin{array}{c}0\\ {}0\end{array}\right) $$ and with the variance-covariance matrices of $$ \left(\begin{array}{cc}{\sigma_{\alpha :b}}^2,& {\sigma}_{\alpha :b,p}\\ {}{\sigma}_{\alpha :b,p},& {\sigma_{\alpha :p}}^2\end{array}\right) $$, $$ \left(\begin{array}{cc}{\sigma_{\beta :b}}^2,& {\sigma}_{\beta :b,p}\\ {}{\sigma}_{\beta :b,p},& {\sigma_{\beta :p}}^2\end{array}\right) $$ and $$ \left(\begin{array}{cc}{\sigma_{\gamma :b}}^2,& {\sigma}_{\gamma :b,p}\\ {}{\sigma}_{\gamma :b,p},& {\sigma_{\gamma :p}}^2\end{array}\right) $$, respectively. The residual error term $$ \left(\begin{array}{c}{\varepsilon}_{(ij)k:b}\\ {}{\varepsilon}_{(ij)k:p}\end{array}\right) $$ with a mean vector of $$ \left(\begin{array}{c}0\\ {}0\end{array}\right) $$ and a variance-covariance matrix of $$ \left(\begin{array}{cc}{\sigma_{\varepsilon :b}}^2,& {\sigma}_{\varepsilon :b,p}\\ {}{\sigma}_{\varepsilon :b,p},& {\sigma_{\varepsilon :p}}^2\end{array}\right) $$ indicates the intra-reader measurement error given a lesion and a reader [16]. The inter-reader measurement error followed a normal distribution with a mean vector of $$ \left(\begin{array}{c}0\\ {}0\end{array}\right) $$ and a variance-covariance matrix of $$ \left(\begin{array}{cc}{\sigma_{\beta :b}}^2+{\sigma_{\gamma :b}}^2+\frac{{\sigma_{\varepsilon :b}}^2}{2},& {\sigma}_{\beta :b,p}+{\sigma}_{\gamma :b,p}+\frac{\sigma_{\varepsilon :b,p}}{2}\\ {}{\sigma}_{\beta :b,p}+{\sigma}_{\gamma :b,p}+\frac{\sigma_{\varepsilon :b,p}}{2},& {\sigma_{\beta :p}}^2+{\sigma_{\gamma :p}}^2+\frac{{\sigma_{\varepsilon :p}}^2}{2}\end{array}\right), $$ as calculated by the variance-covariance component estimates of random effects and residuals to represent deviation in measurements by several readers [13]. Posterior distributions of the variance-covariance components, the parameters of random effects, and the residual errors were obtained using the Markov-chain Monte Carlo method in a Bayesian framework with non-informative priors [17]. Modeling was performed separately for the longest-diameter data and the shortest-diameter data. The modeling process was run in the software R [18].

### Simulation

We simulated a situation in which the baseline and post-treatment diameters of a tumor burden observed in the first reading were re-measured by the same reader or another reader in order to obtain the probability that the change would be classified as a certain tumor response from the re-measured tumor burden sizes. In order to facilitate the simulation, we established artificial datasets of the observed tumor burden sizes and their hypothetical second measurement values using the estimated distributions of intra- or inter-measurement errors. These datasets were used to calculate the probability that the second measurement would be designated as a certain tumor response. This was performed repeatedly for various scenarios. The simulation process was as follows:

- Construction of an artificial dataset of 100 tumor burdens with the same number of target lesions:

When the tumor burden had a single target lesion (*n* = 1), we let ${Y}_{b_1}$ and ${Y}_{p_1}$ be the baseline size and post-treatment size of a lesion, respectively. The lesion size itself became the tumor burden. If we assume that a *c* percent change of the tumor burden occurred, then ${Y}_{p_1}$ is equal to ${Y}_{b_1}+c{Y}_{b_1}$.

When there was a set of tumor burdens having two or more target lesions (*n* ≥ 2), we let ${Y}_{b_x}$ and ${Y}_{p_x}$ be the corresponding baseline size and post-treatment size of the x ^th^ lesion in a tumor burden and let ${\sum}_{x=1}^n{Y}_{b_x}$ and ${\sum}_{x=1}^n{Y}_{p_x}$ therefore correspond to the baseline and post-treatment tumor burden, respectively. If we assume that a *c* percent change of the tumor burden occurred, then ${\sum}_{x=1}^n{Y}_{p_x}$ is equal to ${\sum}_{x=1}^n{Y}_{b_x}+c{\sum}_{x=1}^n{Y}_{b_x}$. The sizes of the lesions in each of the 100 tumor burdens at baseline were generated from a log-normal distribution acquired empirically from the lesion sizes in the original dataset [19]. Because the target lesions within a tumor burden do not change uniformly after treatment, the percent change (*c*_*x*_) of the x ^th^ lesion in a tumor burden was randomly determined from a normal distribution with a mean of *c* and a certain variance, with the restriction that $\sum \limits_{x=1}^n{c}_x{Y}_{bx}$ was equal to $c{\sum}_{x=1}^n{Y}_{b_x}$. The estimated variances using tumor burdens with multiple target lesions from the longest-diameter data and the short-axis diameter data were 0.17 and 0.10, respectively.

(b) Generation of hypothetical second assessments in the baseline and post-treatment phases:

We generated measurement errors $\left({\epsilon}_{b_x},{\epsilon}_{p_x}\right)$ of the *x*^th^ lesion in a burden in the two phases together using the distribution estimated in the previous modeling step. We squared the sum of the square root of the first measurement value produced at (a) and the error at each phase to generate the hypothetical second measurement values of the x^th^ lesions, $\left({\left(\sqrt{Y_{b_x}}+{\epsilon}_{b_x}\right)}^2,{\left(\sqrt{Y_{p_x}}+{\epsilon}_{p_x}\right)}^2\right)=\left({Y}_{b_x}^{\prime },{Y}_{p_x}^{\prime}\right)$. For all lesions in a tumor burden, the above procedure was performed to produce the hypothetical second assessments of the burden, ${\sum}_{x=1}^n{Y_{b_x}}^{\prime }$ and ${\sum}_{x=1}^n{Y_{p_x}}^{\prime }$.

(c) Calculation of the probability of designating a certain tumor response at the second assessment for a given first assessment:

We estimated the probabilities as proportions that the simulated percent changes would be designated as an objective response (i.e., complete or partial response), $\mathrm{P}\left[\frac{\sum_{x=1}^n{Y_{p_x}}^{\prime }-{\sum}_{x=1}^n{Y_{b_x}}^{\prime }}{\sum_{x=1}^n{Y_{b_x}}^{\prime }}\le -0.3\right],$ or progression $, \mathrm{P}\left[\frac{\sum_{x=1}^n{Y_{p_x}}^{\prime }-{\sum}_{x=1}^n{Y_{b_x}}^{\prime }}{\sum_{x=1}^n{Y_{b_x}}^{\prime }}\ge 0.2\right]$.

(d) Producing medians of the probability:

The above process from (a) to (c) was iterated 100 times, using randomly extracted values from the posterior distributions of the variance-covariance component parameters to produce the medians of the estimated probabilities for specific designations.

**Fig. 2:**
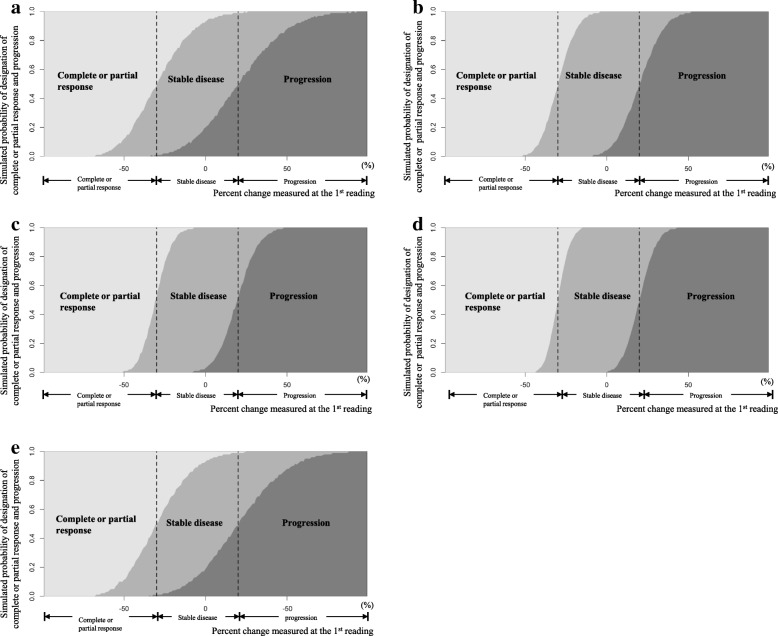
Simulated probability curves of the designation of complete or partial response or progression according to the percent change at the first reading. **a** Simulated probability function when the tumor burden of a 30-mm single target lesion is re-measured by another reader on the long axis. **b** Simulated probability function when the tumor burden of a 120-mm single target lesion is re-measured by another reader on the long axis **c**. Simulated probability function when a tumor burden consisting of four target lesions is re-measured by another reader on the long axis. **d** Simulated probability function when the tumor burden of a 30-mm single target lesion is re-measured by the same reader on the long axis. **e** Simulated probability function when the tumor burden of a 30-mm single target lesion is re-measured by another reader on the short axis

(e) Performing the above simulation processes for different scenarios:

A series of simulation processes was performed for various scenarios, assuming the following factors: different numbers of target lesions (1 ≤ *n* ≤ 5) and different constitutions of the solid lesions (long-axis diameter, up to 5) and lymph nodes (short-axis diameter, up to 2) in the tumor burden; various baseline sizes of the lesion diameter when a single target lesion (*n* = 1) was considered (the ranges of the long- and short-axis diameters were 10–150 mm and 10–80 mm, respectively, and the intervals were 1 mm); different percent changes, − 1.0 ≤ *c* ≤ 1.0 (the interval was 0.01); and within-reader variability or between-reader variability. The distribution of the measurement error was set according to whether a single reader or multiple readers reassessed the lesion size and whether the target lesion was a solid lesion or lymph node. The resulting probability curves from the simulations are exemplified in Fig. 2 for five specific scenarios, with each curve representing the probability for each categorization depending on the scale of percent change measured upon the second assessment.

The above simulation processes were conducted in R software [18].

### Evaluation algorithm

**Fig. 3:**
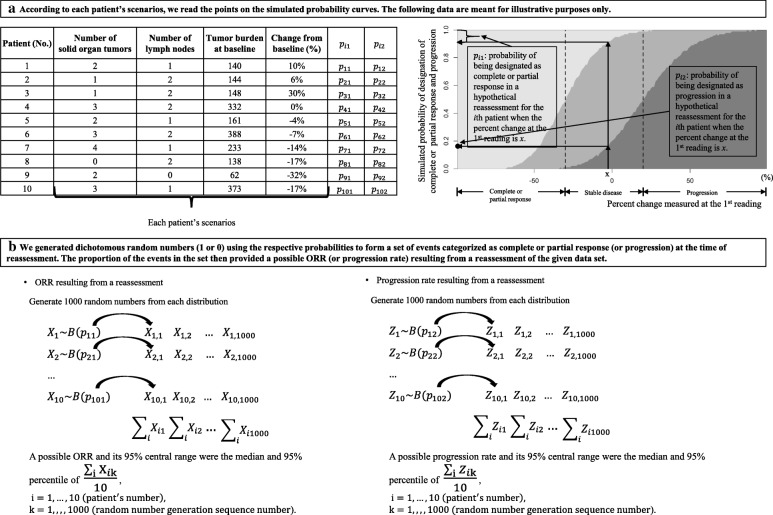
Evaluation algorithm

A diagram of the algorithm is shown in Fig. 3. The algorithm to evaluate the impact of measurement variability on the ORR at the level of a trial features the probabilities calculated in the simulation step. For the trial data of each patient, the probability that the patient’s tumor burden with a reported percent change would be designated as a complete or partial response in a hypothetical reassessment can be obtained (step (a) in Fig. 3). The event that a complete or partial response was declared at the second assessment was a random variable from a Bernoulli distribution with the respective probability. From the given data, we generated dichotomous random numbers (1 or 0) using the corresponding probabilities to form a set of events for determinations of complete or partial response at the time of reassessment (step (b) in Fig. 3). The proportion of the events in the set then provided a possible ORR resulting from a reassessment of the given dataset. By repeating the trial 1000 times, a range of simulated results was produced, with a 95% central range of ORRs determined in the reassessment.

The reliability of the progression rate can be assessed in the same way. However, if patients experience unequivocal radiologic progression, symptomatic progression, or death, a definitive probability of 1 is assigned for progression at the second assessment, as such cases are not affected by measurement variability.

This algorithm was implemented on a website using the Python programming language (Python Software Foundation, https://www.python.org/) [20].

### Validation of the evaluation algorithm using a real dataset

In order to validate the estimated distribution of measurement error from modeling, for each tumor burden in the validation dataset, we calculated the 95% central range of hypothetical results of the second readings. The coverage level was calculated as the proportion of the 95% central ranges that covered the actual burden sizes measured by the second radiologist. For the validation dataset, we produced the 95% central range of simulated ORRs that we could expect from a reassessment using the evaluation tool. We repeated this process using 100 re-sampled datasets drawn by random sampling with replacement from the original dataset, and the coverage level was calculated as the proportion of the 95% central ranges that covered the actually observed ORRs from the second reading. We considered that a coverage level close to 95% was indicative of validity [21].

### Simulation studies

To investigate the impact of different characteristics of data on the reproducibility of trial results, we generated sets of 50 solid tumor burdens at baseline and post-treatment with different characteristics. For simplicity, we only considered single target lesions (i.e., that the tumor burden was the lesion size). Simulation factors were considered for different observed ORR results, different sizes of tumor burden at baseline, and different patterns of the resulting percent change after treatment. We plotted the 95% confidence interval (CI) of the observed ORR and the 95% central range of ORRs obtained from re-assessment by a different observer for each combination of characteristics.

We conducted another series of simulations with similarly created datasets with different characteristics, but in which the true ORR was assumed to be known, and generated trial datasets that each had an observed ORR as an estimate from the known ORR. For each simulated trial dataset, we calculated the point estimate of the ORR based on the generated data as an observation, and plotted its 95% CI and the 95% central range of the ORRs under the assumption that another radiologist would reassess the tumor responses. We analyzed the coverage pattern.

## Results

### Demonstration of the web tool

**Fig. 4:**
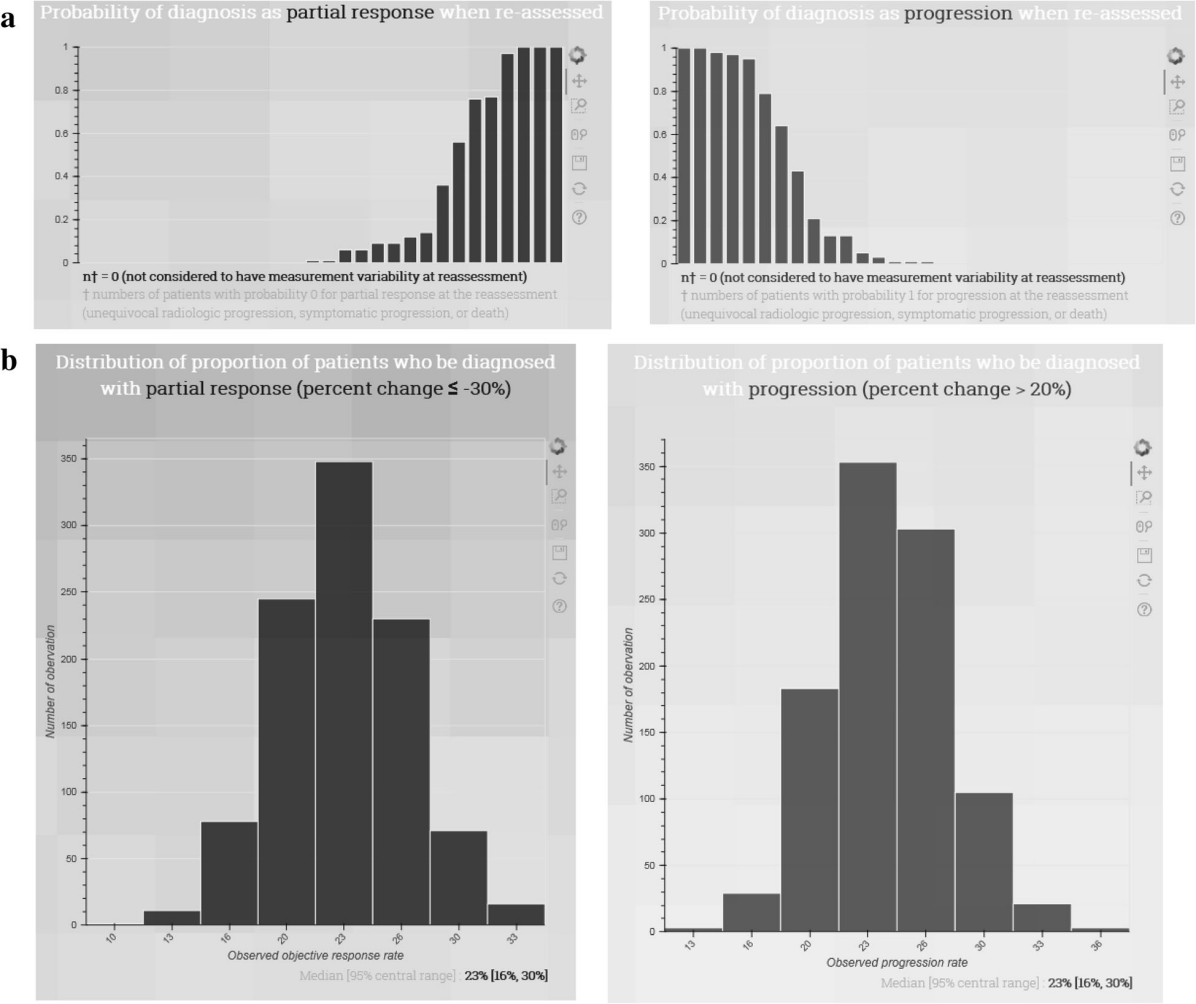
Snapshots of the web tool (response to 1st reviewer’s 5th comment). **a** The probability plots of diagnosis as complete or partial response (left) and progression (right) when re-assessed according to patient ID. Each bar represents one patient. **b** The distribution plots of the simulated objective response rates (left) and progression rates (right) determined in the reassessment. For each distribution, the median and the 95% central range are presented

An algorithm for calculating the 95% central range of expected ORRs (or progression rates) in a repeated trial was developed, and it is available online as a web tool (http://studyanalysis2017.pythonanywhere.com/). For usage of the tool, a dataset containing the patient ID, organ information (solid organ or lymph node), lesion size at baseline, and lesion size at post-treatment should be uploaded, and further information about the dataset should be added, including the study name, treatment name, and the number of enrolled patients with unequivocal radiologic progression, symptomatic progression, or death. On the webpage, the uploaded data are confirmed and processed, yielding the number of solid organ tumors, the number of lymph nodes, the percent change (%), the tumor burden at baseline (mm), and the tumor burden at post-treatment (mm). According to whether the same or another radiologist were to reassess the tumor response, the probability of being diagnosed with a complete or partial response (or progression) upon a reassessment of each patient is presented (Fig. 4(a)). Finally, the 95% central range of the ORRs and 95% central range of the progression rates determined in the reassessment are shown (Fig. 4(b)).

### Validation results of the estimated distributions of measurement errors and the evaluation algorithm

**Fig. 5:**
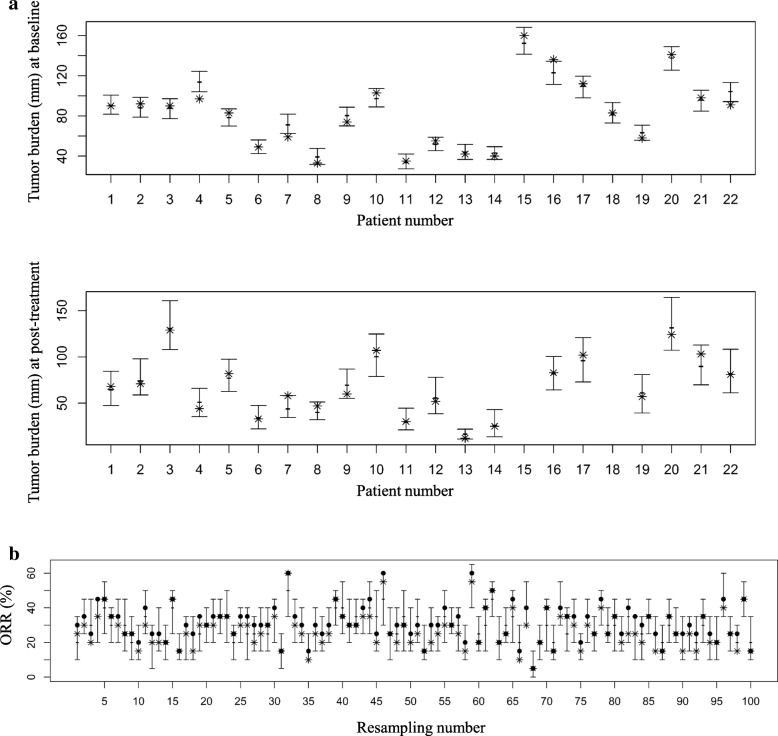
Interval plots of results of the validation and actual measurements. **a** Tumor burden size per patient at baseline (above) and post-treatment (below). The intervals indicate the 95% central ranges of the simulated burden sizes. The stars indicate the tumor burden sizes re-measured by the second radiologist. **b** ORR per resampling. The intervals indicate the 95% central ranges of the objective response rates (ORRs) from the simulation. The circles and stars indicate the ORRs measured by the first radiologist and the second radiologist, respectively

In the validation of the estimated distribution of measurement errors, the actual second measurement value was located within the 95% central range of the hypothetical second readings in 19 of 22 and 20 of 20 burdens at baseline and post-treatment, respectively (Fig. 5(a)). Therefore, the coverage level was 93%. Concerning the results of the validation of the evaluation algorithm, for the 100 resampled datasets, the ORR resulting from a real second assessment lay in the 95% central range of simulated ORRs in all datasets (Fig. 5(b)). The coverage level was 100%.

### Simulation studies

**Fig. 6:**
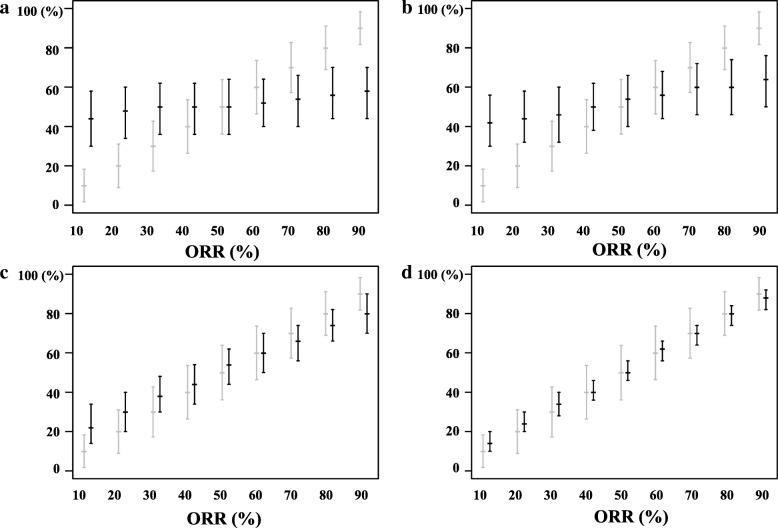
95% confidence intervals and 95% central ranges depending on the different observed results and the characteristics of the trial data. **a** When the baseline lesion size is 30 mm and the distribution of percent change is −30 ± *N*(0, 5^2^) (**b**) When the baseline lesion size is 100 mm and the distribution of percent change is −30±*N* (0, 5^2^) (**c**) When the baseline lesion size is 30 mm and the distribution of percent change is −30 ± *N*(20, 5^2^) (**d**) When the baseline lesion size is 100 mm and the distribution of percent change is −30 ± *N*(20, 5^2^). Gray lines: the observed objective response rate (ORR) and 95% confidence interval; Black lines: median and 95% central range from the tool

For the trial data with a smaller lesion size at baseline, the 95% central ranges of the re-assessed ORRs tended to be wider ((a) versus (b) and (c) versus (d) in Fig. 6). For the trial data where the occurred percent changes of tumor burdens were distributed closely around the response cutoff value of − 30%, the 95% central ranges of the re-assessed ORRs tended to be wider, and did not coincide with the 95% CI in many cases apart from when the observed ORRs were close to 50% ((a) versus (c) and (b) versus (d) in Fig. 6). High reproducibility was observed when the baseline lesion size was large and the occurred percent changes were distributed largely away from the cutoff value (Fig. 6 (d)).

The second set of simulation studies also showed a similar pattern (Additional file 2: Figure S1, Additional file 3: Figure S2, and Additional file 4: Figure S3). The 95% CIs tended to coincide well with the 95% central ranges of the re-assessed results of ORRs when the assumed true ORR was 50%, regardless of the baseline burden sizes and percent changes. When the assumed ORRs were smaller or greater than 50% by a difference of 30%, the tendency for overlap depended on the distribution of percent changes, and in particular how closely they were aggregated to the cutoff. When the percent changes were distributed closely to − 30%, the degree of overlap was small, indicating that the data were very vulnerable to measurement variability in assessing tumor response. When every other condition was fixed, larger baseline burden sizes resulted in larger overlap. Whether the 95% central range covered a point estimate of ORR also demonstrated a similar pattern.

## Discussion

In oncology, treatment response is determined based on radiologic assessments of the tumor burden, which are subject to measurement variability. It has not been possible to assess the degree to which the resulting ORRs and progression rate in specific trials are robust against measurement variability. In this study, we developed an algorithm for quantifying the impact of measurement variability on the ORR and progression rate from a specific trial, as well as a web tool for implementing the algorithm. We presented the sequence of steps used to develop the algorithm, including a method of modeling measurement error, a method of calculating the probability representing the effect of measurement error on determination of treatment response, and the process of applying the final algorithm to evaluate the impact of measurement variability on the results from a trial.

Our hierarchical linear mixed model was constructed with the goal of estimating the variance components capturing measurement variability as the remaining variation after accounting for other specific sources of variation. Although the data structure suggested that lesions were nested with patients, because the variation among the patient-specific measurements was in fact part of the variation of the lesion-specific measurements, it was unnecessary to separate these in the model because this nesting did not influence the remaining variations. The estimated distributions of intra-reader and inter-reader measurement errors did not change depending on whether or not the patient effect was added to the model.

Square root-transformed data were used as the response variable in our model to approximate normality and to account for the measurement size-dependency of measurement errors. Furthermore, we assumed that the inherent characteristics of a lesion would have a similar effect on measurement errors at baseline and at post-treatment. We thus used a bivariate mixed-effects model to account for the correlation between measurement errors before and after treatment.

There was a small proportion of outlying data observed in LOAs presented with a Bland-Altman plot. By examining detailed images of the outlying data, it was judged that those outlying results originated solely from misperceptions of the boundaries of target lesions due to abutting non-malignant pathology such as atelectasis or the misunderstanding of target lesions, and such cases exceeded the range of measurement error, which rarely occurs in oncology practice. For this reason, we decided to trim off the outlying 5% of the data.

Our primary interest was to investigate the degree to which the original conclusion of a specific clinical trial would be stable against measurement variability if the data of the given trial were reassessed. We used a framework focusing on early-phase clinical trials that draw conclusions based on the ORR and the percentage of patients who achieve complete or partial response. Complete response was therefore dealt with as part of the definition of ORR rather than by evaluating it separately. The results from the simulation step demonstrated that for extreme negative values of *c* (with *c* =  − 1 defined as a complete response) the probability of designating a complete or partial response at the second assessment was almost 1, as seen in Fig. 2. That is, in such cases, measurement variability may have a minimal effect on the response determination. The evaluation algorithm took this into account in the calculation.

In the simulation process to obtain the probability of a complete or partial response, the probability distribution was considered through an iterative procedure by using randomly extracted values from the posterior distributions of the variance-covariance component parameters. Since the posterior distributions were leptokurtic and the variance of those probabilities obtained from the iterative processes was very small, we determined that use of a fixed probability by the median value would be sufficient for ensuring simplicity of the evaluation algorithm.

The 95% central range of the ORR can be used as an interpretable indicator of the robustness of the originally reported ORR against measurement variability. A narrow 95% central range indicates a high reproducibility of the ORR despite measurement variability, as manifested by close agreement between the results of categorization assessed by the same reader (performing a repeat reading) or different readers, accounting for measurement variability. However, the 95% central range of the re-assessed ORRs should be interpreted differently from the 95% CI of the observed ORR. The 95% CI deals with the uncertainty against sampling errors in the estimation of the ORR, while the 95% central range of the ORR deals with the reproducibility of the observed ORR against measurement variability in a given sample. These two measures shed light on different aspects of uncertainty of an observed result from a given clinical trial. It is therefore advised that researchers should more carefully consider the robustness of their usual inferences based on the CI when considering potential reassessments of the same tumor burdens by themselves or by other observers. We also demonstrated that the 95% central range of reproduced ORRs could be different from the 95% CI of the originally observed ORR depending on different characteristics of the data in terms of the baseline tumor burden and percent changes through simulation studies.

When drawing a conclusion based on a RECIST-based response from clinical trial data, the observed ORR is typically presented as a point estimate with a 95% central interval and a waterfall plot. Those presentations do not suggest any information on the composition of tumor burdens with a specific post-treatment percent change, or in other words, the degree to which the observed ORR is robust against measurement variability. Thus, it is impossible for clinical trialists to explore this aspect of robustness, unless a repeated evaluation of tumor responses in the particular trial dataset is actually undertaken, such as a blinded independent central review, which is only applied in phase 2 or 3 trials to a limited extent in practice due to considerable requirements in terms of time and resources. The algorithmic tool presented herein can be considered a pragmatic, practical alternative to reassessment exercises by the original reader or another reader.

Simulation studies have provided some useful insights into how the 95% central range can be practically interpreted. A 95% central range of reassessed ORRs can be a useful tool for evaluating the robustness of an observed ORR against measurement variability by assessing the degree to which the 95% central range overlaps the 95% CI of the observed ORR. When the trial dataset primarily consisted of tumor burdens with tumor responses susceptible to measurement variability (i.e., tumor responses with post-treatment percent changes close to the cutoff of − 30%, with smaller tumor burdens), the 95% central range was wider, suggesting that the ORR could be just 50% (i.e., only a half chance of response), regardless of the 95% confidence interval of the observed ORR. When the trial dataset primarily consisted of tumor burdens with tumor responses insensitive to measurement variability (i.e., tumor responses with post-treatment percent changes further from − 30%, with larger tumor burdens), the 95% central range was narrower and coincided closely with the 95% CI. If the 95% CI and the 95% central range of the observed ORR rarely overlap and are apart from each other, clinical trialists can conclude that the observed ORR is unlikely to be reproducible if the trial data are repeatedly assessed. If the 95% CI of the observed ORR entirely covers the 95% central range of re-assessed ORRs, clinical trialists may conclude that the observed ORR is likely to be highly reproducible even if repeatedly assessed, and the impact of measurement variability is negligible.

This study has several limitations. First, since the algorithm was developed based on data composed of CT image measurements from a specific oncology trial of advanced SCLC, validation using external data was essential. The algorithm worked properly in an external dataset of images obtained from patients with refractory SCLC. However, further research should be undertaken to examine the generalizability of the algorithm by using clinical trial data on different types of cancer. Second, the characteristics of a lesion (i.e., border irregularity and conglomeration) may affect estimations of the distribution of the measurement error. We were unable to perform a subgroup analysis according to these two factors due to a lack of data. However, as shown in the validation results for the distribution of measurement errors, the simulated second burden size was adequate for replicating the actual second size. Third, we included long-axis diameters for lymph nodes in the dataset for developing a long-axis diameter model. However, the RECIST guideline (version 1.1) recommends measuring the short-axis diameter for lymph nodes. Nevertheless, the procedure is still acceptable, as the long-axis diameter of lymph nodes was used for representing the tumor burden in the previous RECIST guideline (version 1.0) [22], and the reason for changing the measurement axis for lymph nodes is that normal lymph nodes can have a measurable size without metastasis and the short-axis diameter is more predictive of metastasis [23]. Finally, in the simulation, when the tumor burden was from a single lesion, various baseline sizes from 10 mm to 150 mm were considered in order to investigate the pattern of the simulated probability curve according to baseline size. When the tumor burden was composed of multiple lesions, we generated lesion sizes for a range of fixed numbers of lesions in a tumor burden, based on an empirical distribution of lesion sizes. This enabled us to account for the number of lesions within a tumor burden, which is closely related to the burden size, but the burden size itself was not considered. Thus, our procedure could be considered to be a rough assessment of the baseline burden size with multiple lesions, but this issue should be further investigated.

## Conclusions

Our validation exercise demonstrated the adequacy of the statistical modeling approach and the utility of the developed algorithm. The web-based tool can be used for the future evaluation of trial results to predict the impact of measurement variability on the results. Quantification of the variation in the ORR or progression rate due to potential measurement variability is essential, and will help inform decisions made on the basis of clinical trial data. Although there are some issues remaining for further elaboration, the demonstration of the process and the findings of this study should provide an important basis for future research in relation to measurement variability, particularly in oncology.

## Additional files

Additional file 1: Formulas of the LOAs on the original scale for the nth root-transformed variables. (DOCX 18 kb)

Additional file 2: **Figure S1.** Observed objective response rates (ORR) with 95% confidence intervals and 95% central ranges from the evaluation tool depending characteristics of simulated data sets in which the true ORR is 20%. (a) when the assumed distributions of baseline tumor burden size and percent change are *LN*(3.55, 0.53^2^) and − 30±*N*(0, 5^2^), respectively. (b) when the assumed distributions of baseline tumor burden size and percent change are *LN*(3.55, 1.22^2^) and − 30±*N*(0, 5^2^), respectively. (c) when the assumed distributions of baseline tumor burden size and percent change are *LN*(3.55, 0.53^2^) and − 30±*N*(0, 20^2^), respectively. (d) when the assumed distributions of baseline tumor burden size and percent change are *LN*(3.55, 1.22^2^) and − 30±*N*(0, 20^2^), respectively. (e) when the assumed distributions of baseline tumor burden size and percent change are *LN*(4.25, 0.53^2^) and − 30±*N*(0, 5^2^), respectively. (f) when the assumed distributions of baseline tumor burden size and percent change are *LN*(3.55, 0.53^2^) and − 30±*N*(20, 5^2^), respectively. (g) when the assumed distributions of baseline tumor burden size and percent change are *LN*(4.25, 0.53^2^) and − 30±*N*(20, 5^2^), respectively. Dashed line: true ORR; Gray lines: The observed ORR and 95% confidence interval; Black lines: median and 95% central range from the tool. (PDF 334 kb)

Additional file 3: **Figure S2.** Observed objective response rates (ORR) with 95% confidence intervals and 95% central ranges from the evaluation tool depending characteristics of simulated data sets in which the true ORR is 50%. (a) when the assumed distributions of baseline tumor burden size and percent change are *LN*(3.55, 0.53^2^) and − 30±*N*(0, 5^2^), respectively. (b) when the assumed distributions of baseline tumor burden size and percent change are *LN*(3.55, 1.22^2^) and − 30±*N*(0, 5^2^), respectively. (c) when the assumed distributions of baseline tumor burden size and percent change are *LN*(3.55, 0.53^2^) and − 30±*N*(0, 20^2^), respectively. (d) when the assumed distributions of baseline tumor burden size and percent change are *LN*(3.55, 1.22^2^) and − 30±*N*(0, 20^2^), respectively. (e) when the assumed distributions of baseline tumor burden size and percent change are *LN*(4.25, 0.53^2^) and − 30±*N*(0, 5^2^), respectively. (f) when the assumed distributions of baseline tumor burden size and percent change are *LN*(3.55, 0.53^2^) and − 30±*N*(20, 5^2^), respectively. (g) when the assumed distributions of baseline tumor burden size and percent change are *LN*(4.25, 0.53^2^) and − 30±*N*(20, 5^2^), respectively. Dashed line: true ORR; Gray lines: the observed ORR and 95% confidence interval; Black lines: median and 95% central range from the tool. (PDF 323 kb)

Additional file 4: **Figure S3.** Observed objective response rates (ORR) with 95% confidence intervals and 95% central ranges from the evaluation tool depending characteristics of simulated data sets in which the true ORR is 80%. (a) when the assumed distributions of baseline tumor burden size and percent change are *LN*(3.55, 0.53^2^) and − 30±*N*(0, 5^2^), respectively. (b) when the assumed distributions of baseline tumor burden size and percent change are *LN*(3.55, 1.22^2^) and − 30±*N*(0, 5^2^), respectively. (c) when the assumed distributions of baseline tumor burden size and percent change are *LN*(3.55, 0.53^2^) and − 30±*N*(0, 20^2^), respectively. (d) when the assumed distributions of baseline tumor burden size and percent change are *L*N(3.55, 1.22^2^) and − 30±*N*(0, 20^2^), respectively. (e) when the assumed distributions of baseline tumor burden size and percent change are *LN*(4.25, 0.53^2^) and − 30±*N*(0, 5^2^), respectively. (f) when the assumed distributions of baseline tumor burden size and percent change are *LN*(3.55, 0.53^2^) and − 30±*N*(20, 5^2^), respectively. (g) when the assumed distributions of baseline tumor burden size and percent change are *LN*(4.25, 0.53^2^) and − 30±*N*(20, 5^2^), respectively. Dashed line: true ORR; Gray lines: the observed ORR and 95% confidence interval; Black lines: median and 95% central range from the tool. (PDF 329 kb)

## Abbreviations

CI: Confidence interval
CT: Computed tomography
IRB: Institutional review board
LOA: Limits-of-agreement
ORR: Objective response rate
RECIST: Response evaluation criteria in solid tumors
