# Supplementary material for: Development of an algorithm for evaluating the impact of measurement variability on response categorization in oncology trials
Source: BMC Med Res Methodol. 2019 May 2;19:90. doi: 10.1186/s12874-019-0727-7 (PMC6498480; doi:10.1186/s12874-019-0727-7)
Supplement: Supplementary file 1 — Formulas of the LOAs on the original scale for the nth root-transformed variables. (DOCX 18 kb) [file 12874_2019_727_MOESM1_ESM.docx]

**Formulas of the LOAs on the original scale for the nth root-transformed variables**

A 95% LOA for the *n*th root transformation of the measurement, $Y^{n} (0<n<1)$, is $0\pm\alpha$, with α$=1.96\cdot SD$ (SD: the standard deviation of the difference between the *n*th root transformed for two measurements per subject). Subsequently, it can be plotted on the original scale in a conventional Bland-Altman plot [1]: $Y_{1}-Y_{2}$ is between $-\frac{1}{n}\alpha\bar{Y}^{1-n}$ and $\frac{1}{n}\alpha\bar{Y}^{1-n}$ for a given $\bar{Y}$ value according to the mean value theorem ($Y_{1}$: the first measurement$, Y_{2}$: the second measurement$, \bar{Y}$: the average of $Y_{1}$ $\mathrm{and}Y_{2}$). For example, when the measurements are transformed with the square root, the intra-reader LOA and the inter-reader LOA on the original scale are $0\pm2\cdot1.96\sqrt{2\sigma_{\varepsilon}^{2}}\cdot\sqrt{\bar{Y}}$ and $0\pm2\cdot1.96\sqrt{2(\sigma_{\beta}^{2}+\sigma_{\gamma}^{2}+\frac{\sigma_{\varepsilon}^{2}}{2})}\cdot\sqrt{\bar{Y}}$, respectively. $\sigma_{\beta}^{2}$, $\sigma_{\gamma}^{2}$, and $\sigma_{\varepsilon}^{2}$ were estimated using a hierarchical linear mixed-effects model including lesions, readers, and the interaction effects of lesions and readers as random effects.

# **References**

1. Euser AM, Dekker FW, le Cessie S: **A practical approach to Bland-Altman plots and variation coefficients for log transformed variables**. *Journal of clinical epidemiology* 2008, **61**(10):978-982.
