# Supplementary figures and images for: Development of an algorithm for evaluating the impact of measurement variability on response categorization in oncology trials
Source: BMC Med Res Methodol. 2019 May 2;19:90. doi: 10.1186/s12874-019-0727-7 (PMC6498480; doi:10.1186/s12874-019-0727-7)

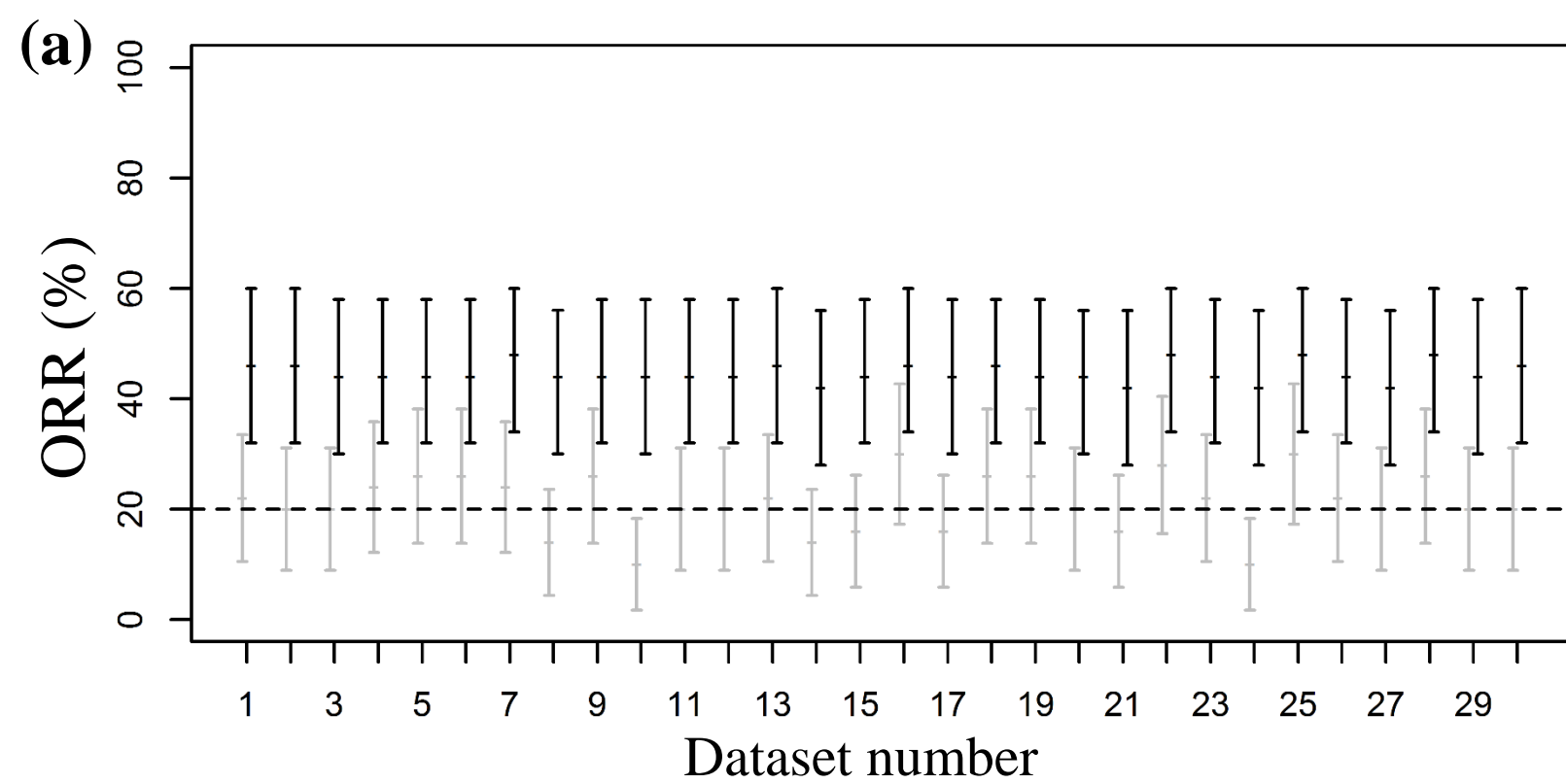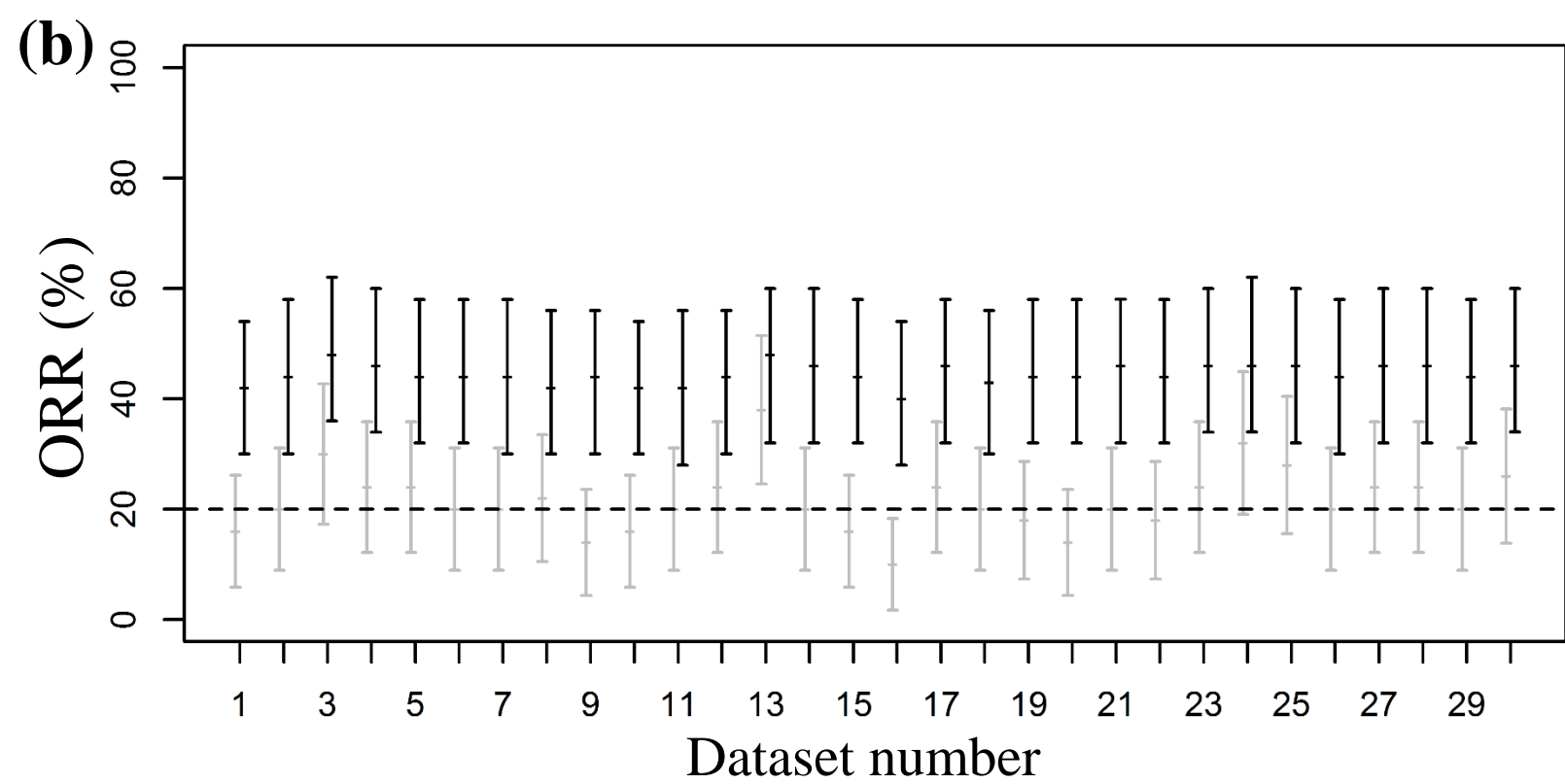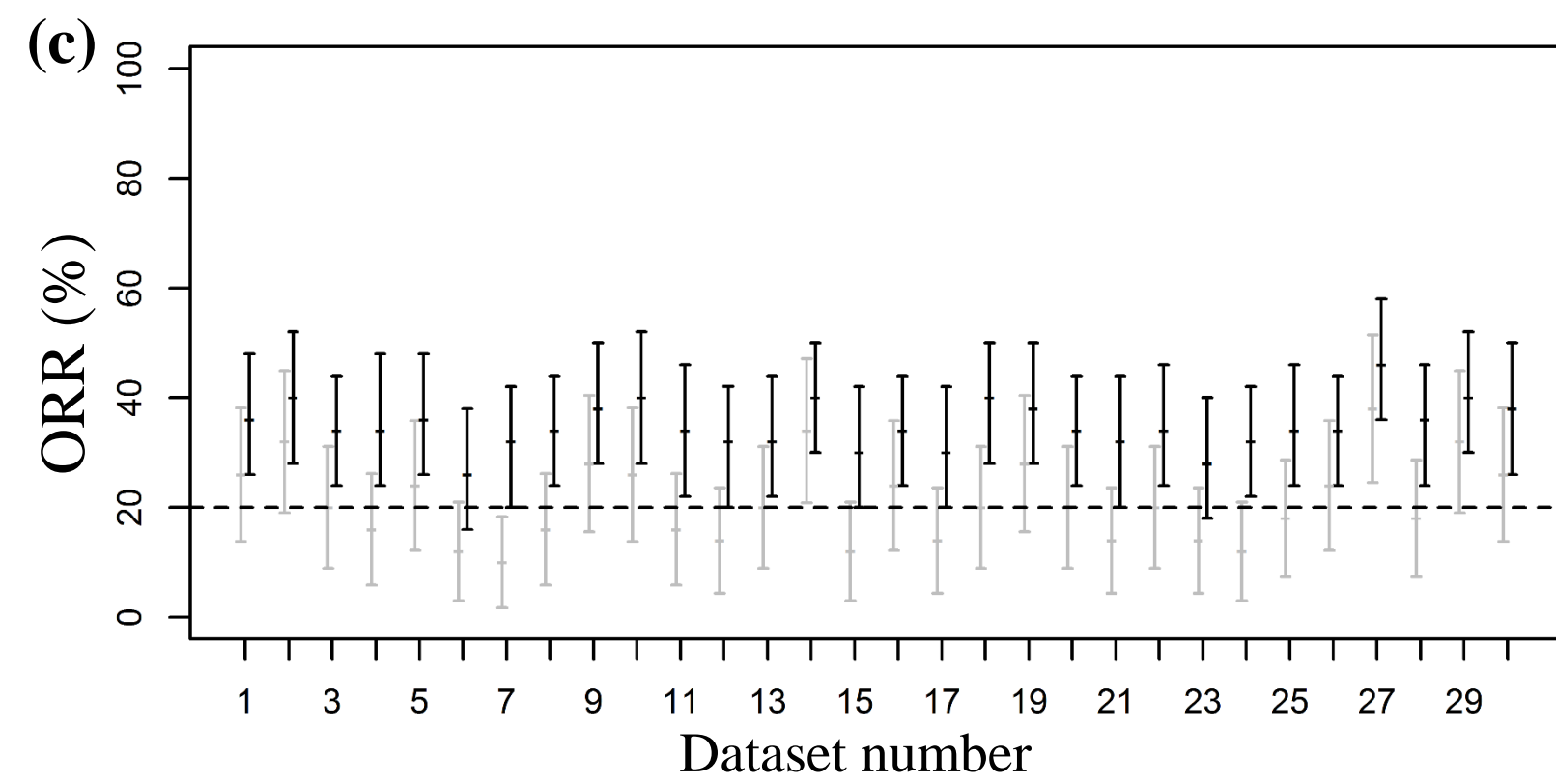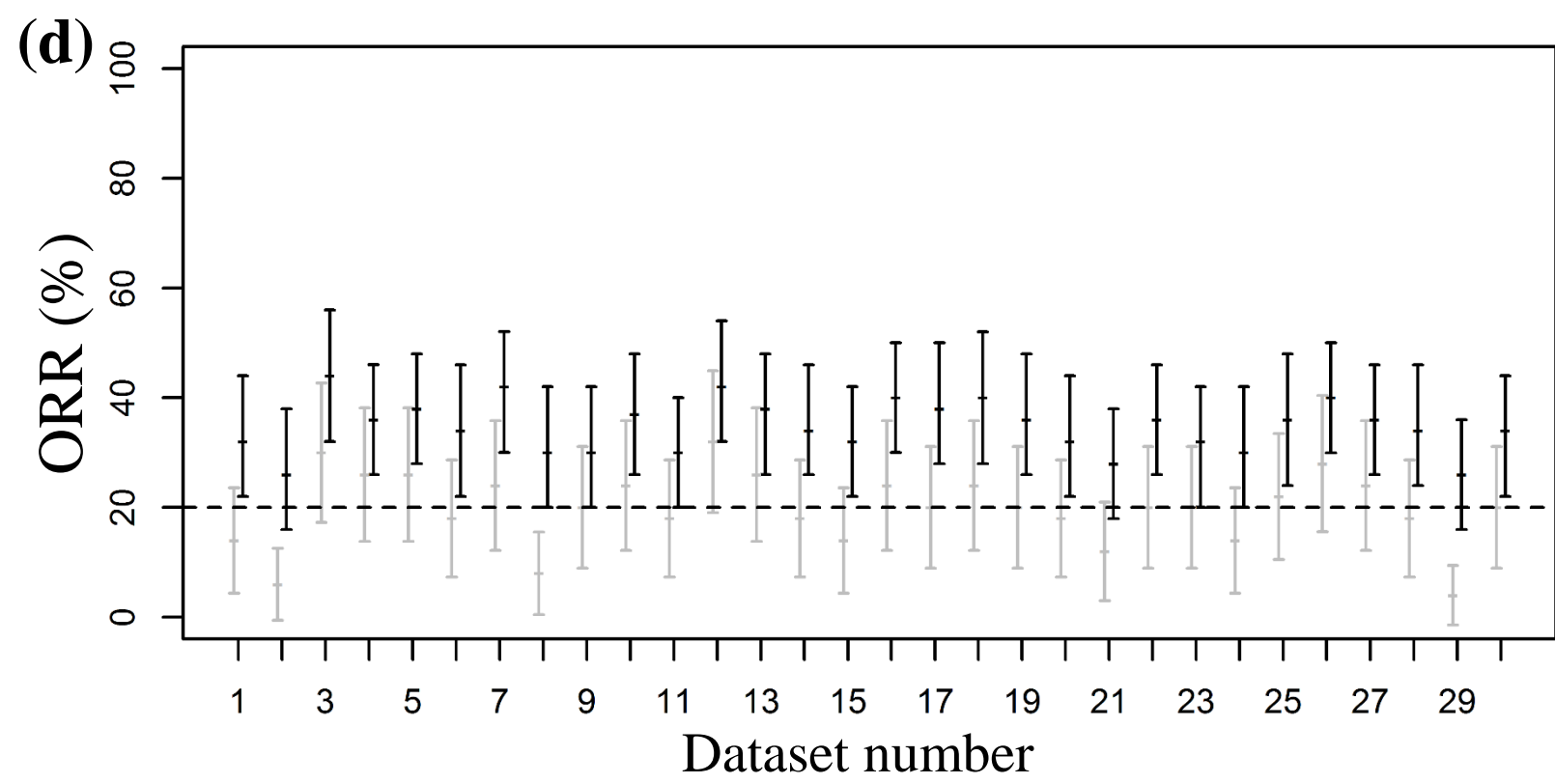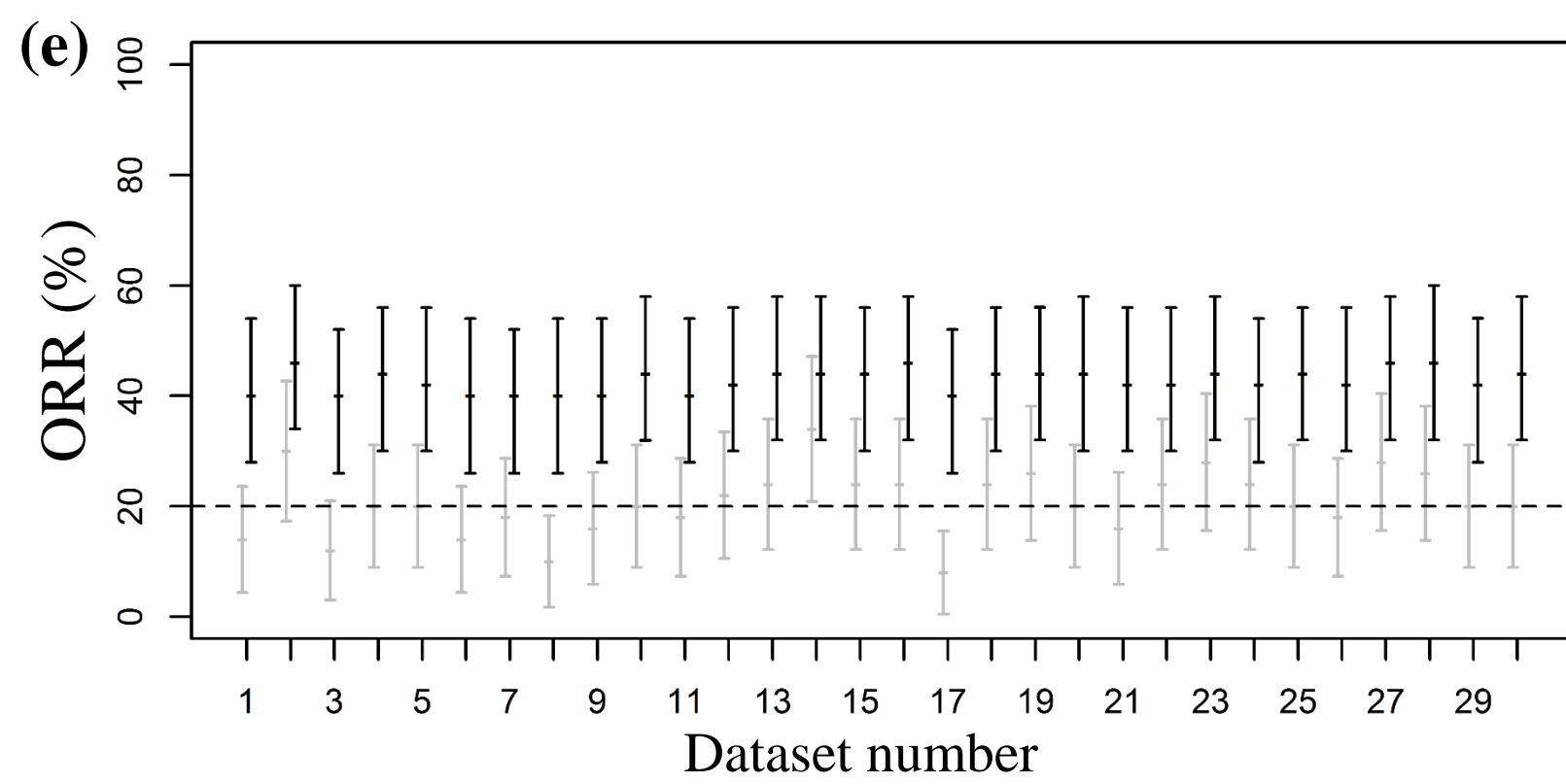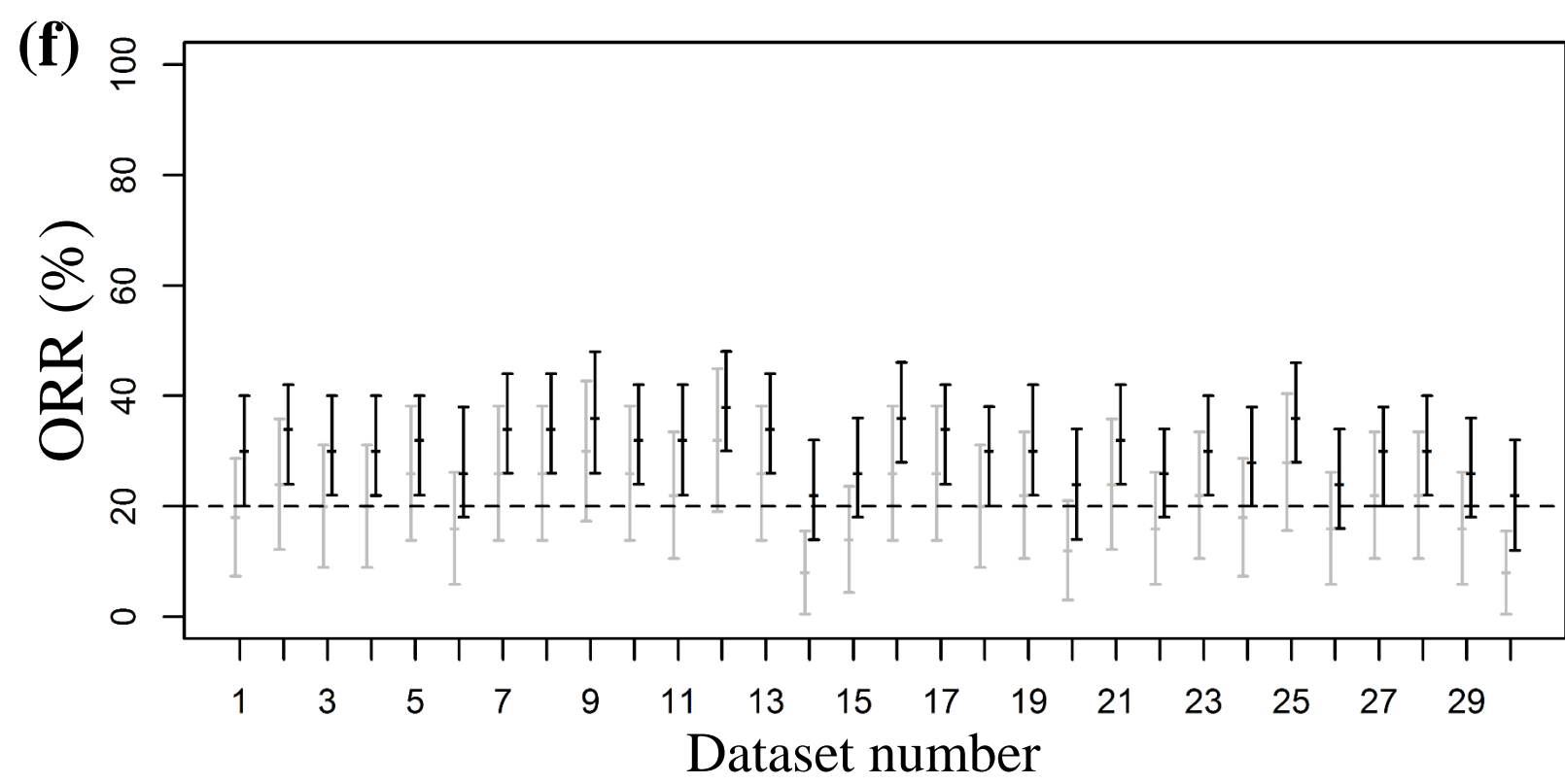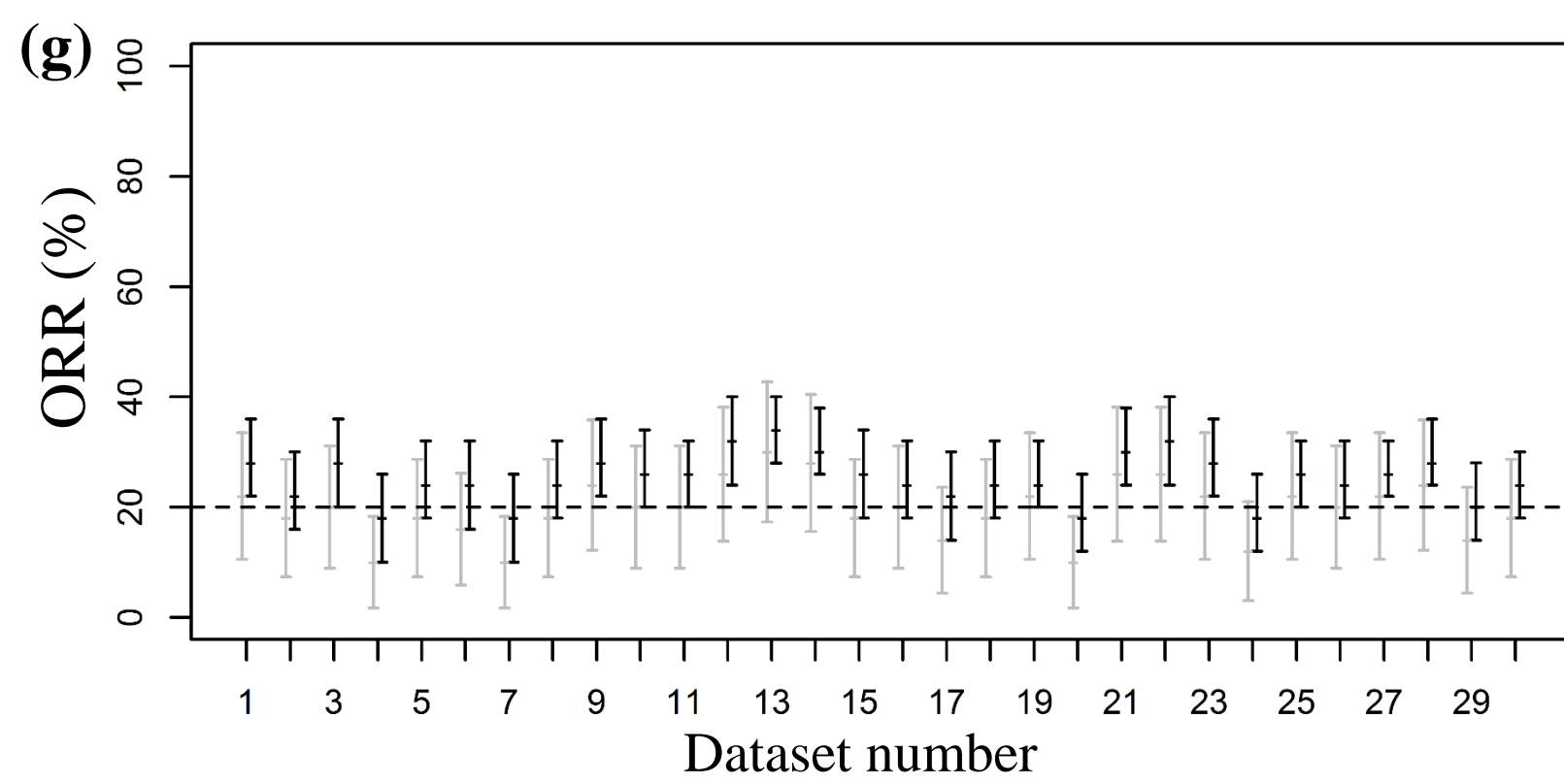

Supplement: Supplementary file 2 — Figure S1. Observed objective response rates (ORR) with 95% confidence intervals and 95% central ranges from the evaluation tool depending characteristics of simulated data sets in which the true ORR is 20%. (a) when the assumed distributions of baseline tumor burden size and percent change are LN(3.55, 0.532) and − 30±N(0, 52), respectively. (b) when the assumed distributions of baseline tumor burden size and percent change are LN(3.55, 1.222) and − 30±N(0, 52), respectively. (c) when the assumed distributions of baseline tumor burden size and percent change are LN(3.55, 0.532) and − 30±N(0, 202), respectively. (d) when the assumed distributions of baseline tumor burden size and percent change are LN(3.55, 1.222) and − 30±N(0, 202), respectively. (e) when the assumed distributions of baseline tumor burden size and percent change are LN(4.25, 0.532) and − 30±N(0, 52), respectively. (f) when the assumed distributions of baseline tumor burden size and percent change are LN(3.55, 0.532) and − 30±N(20, 52), respectively. (g) when the assumed distributions of baseline tumor burden size and percent change are LN(4.25, 0.532) and − 30±N(20, 52), respectively. Dashed line: true ORR; Gray lines: The observed ORR and 95% confidence interval; Black lines: median and 95% central range from the tool. (PDF 334 kb) [file 12874_2019_727_MOESM2_ESM.pdf]

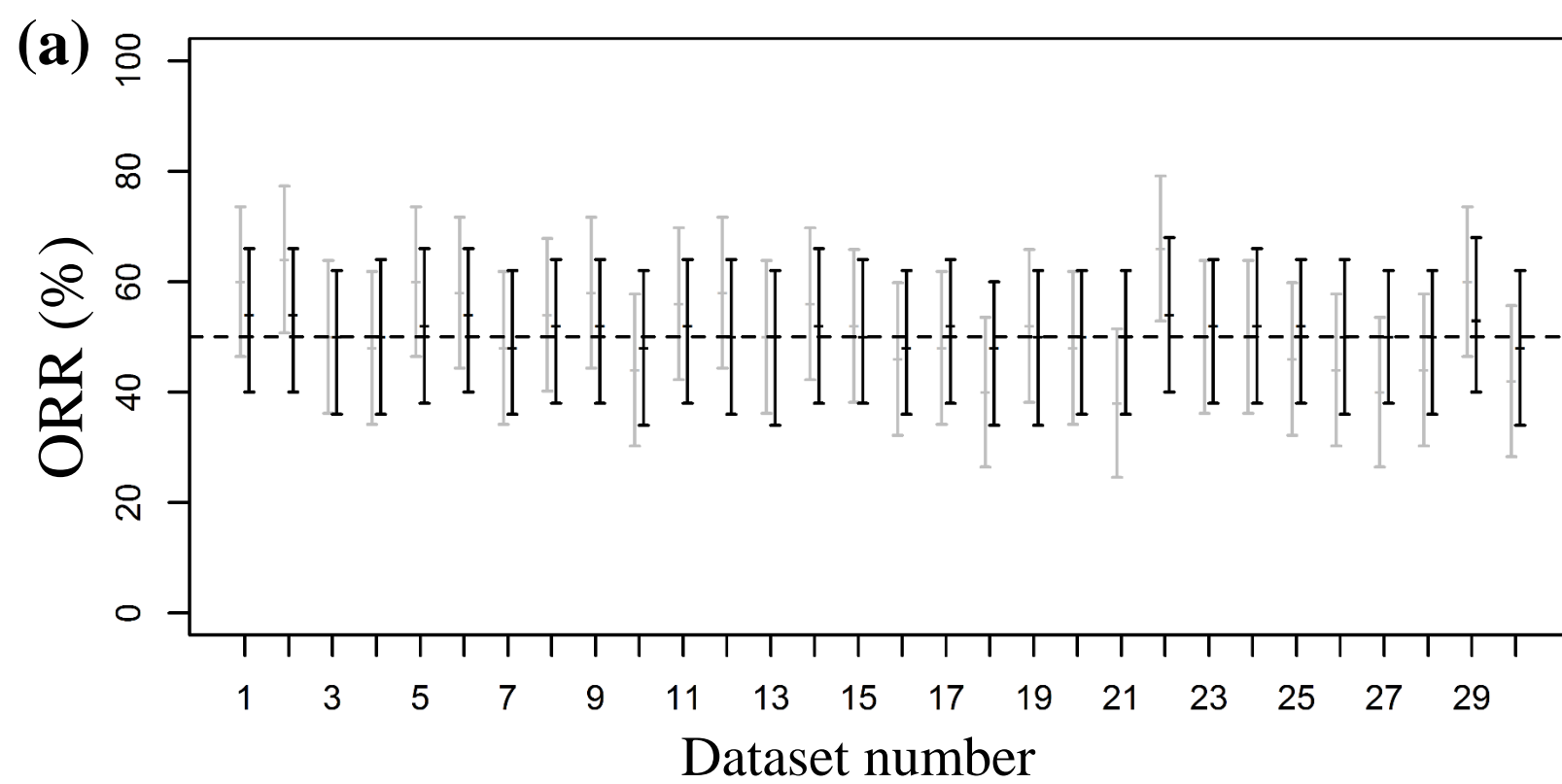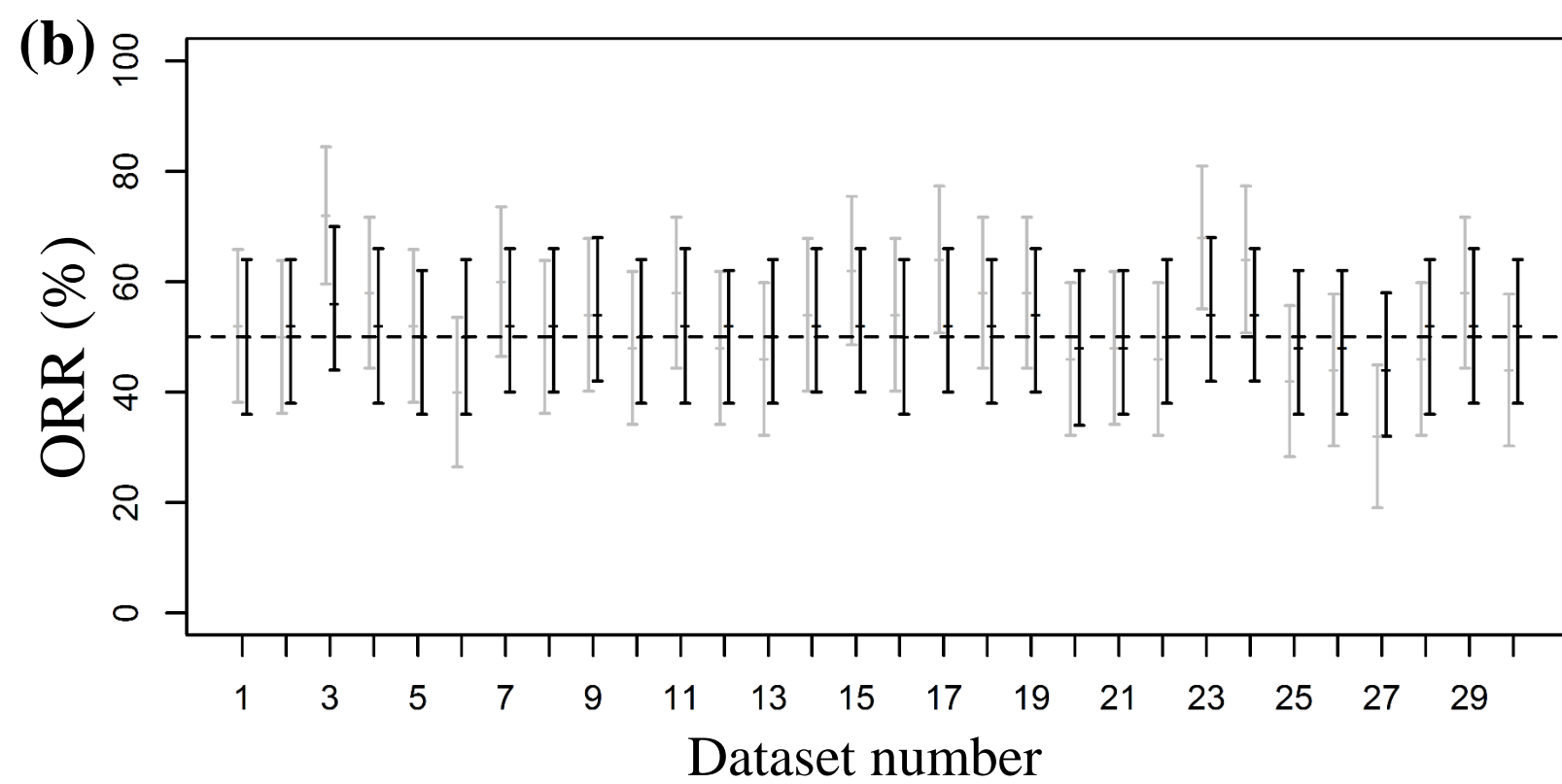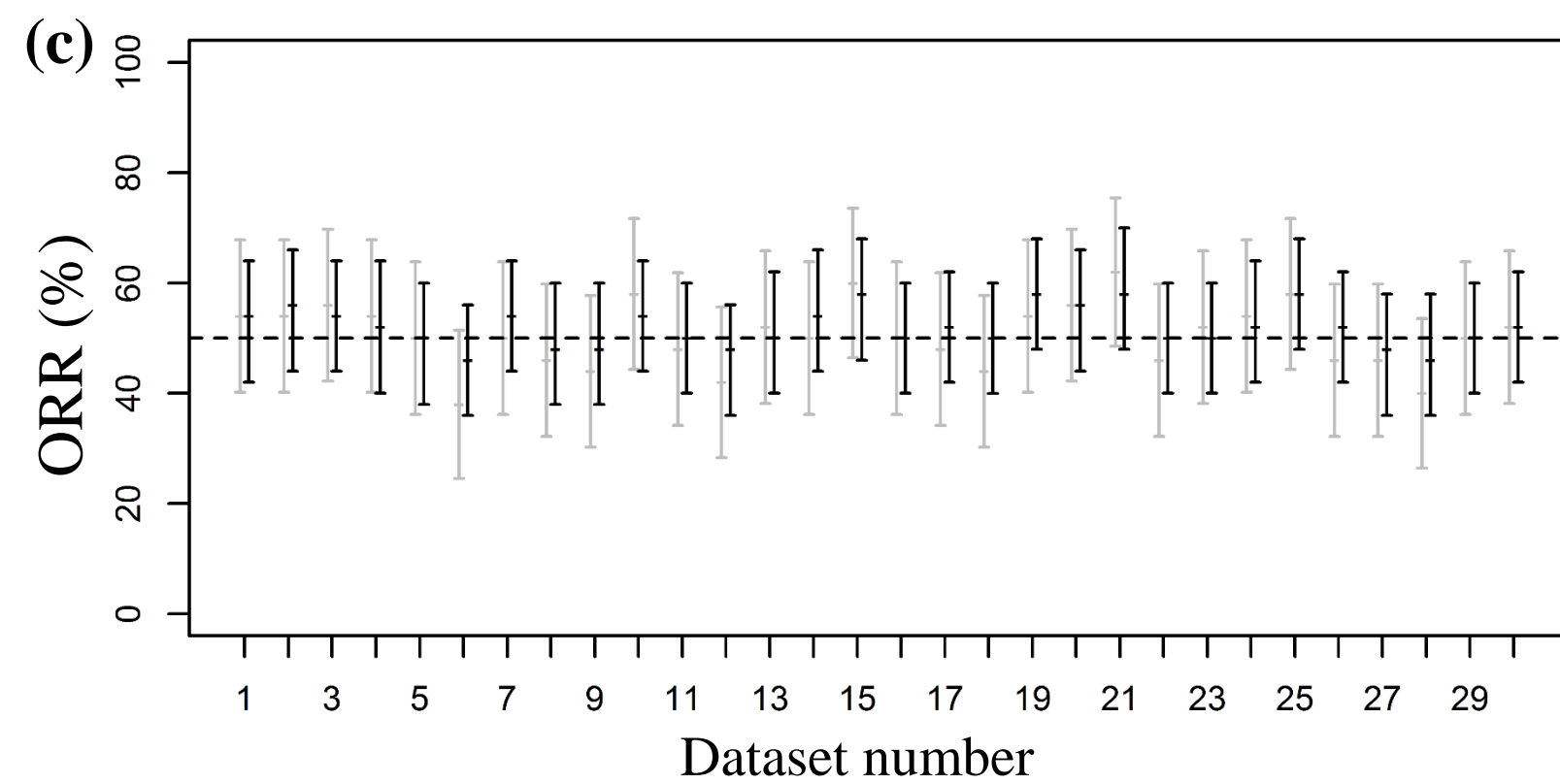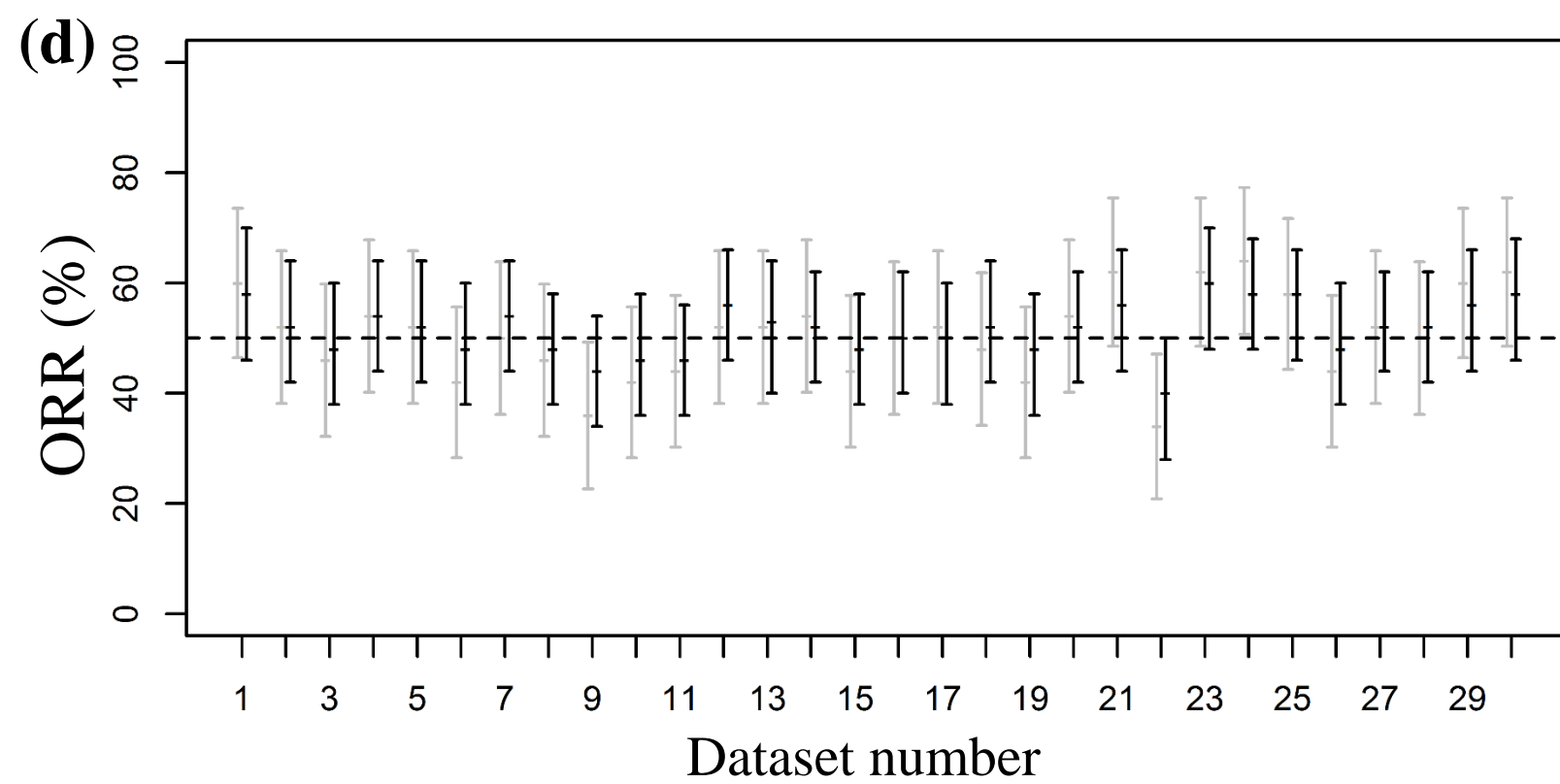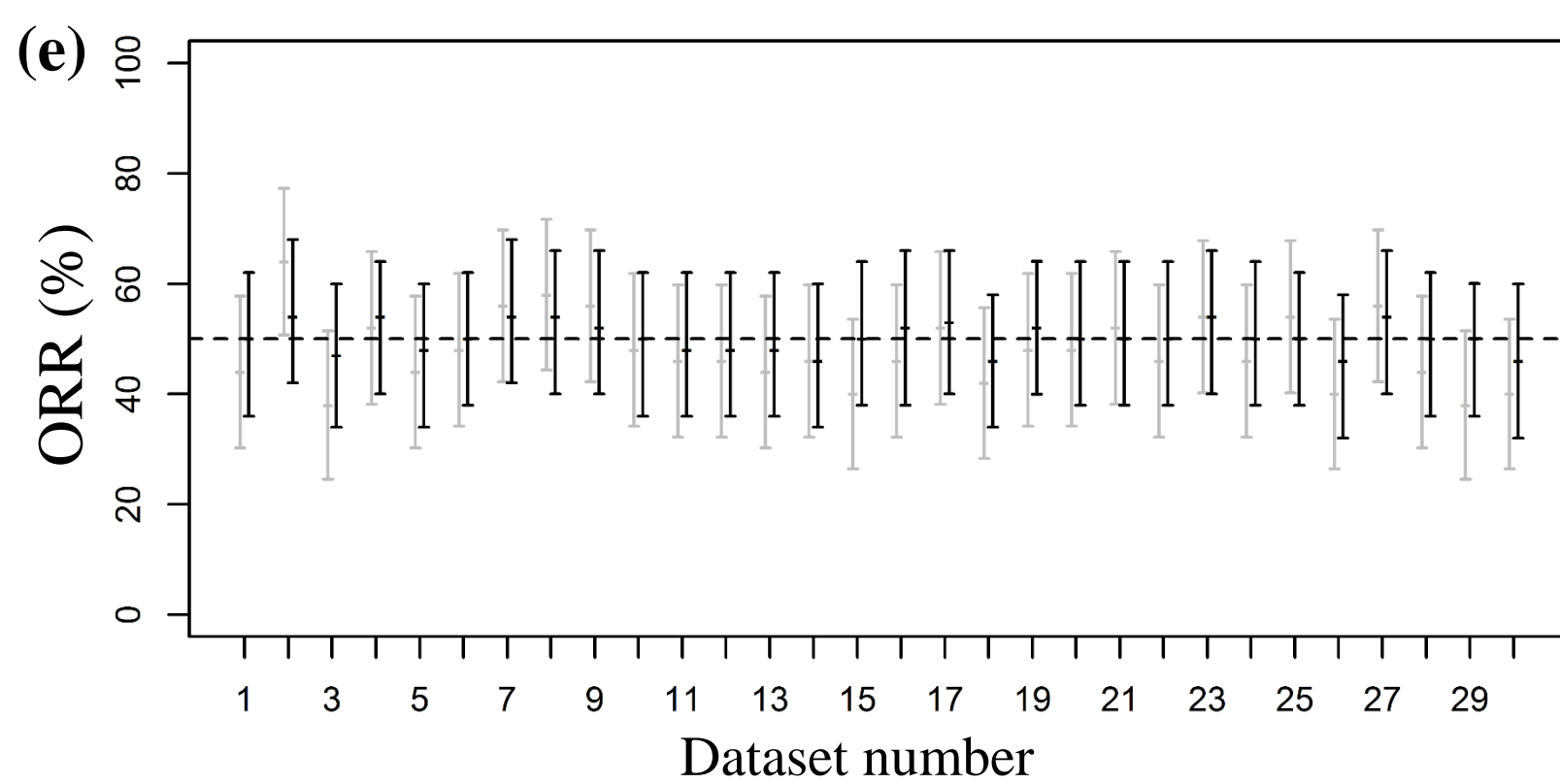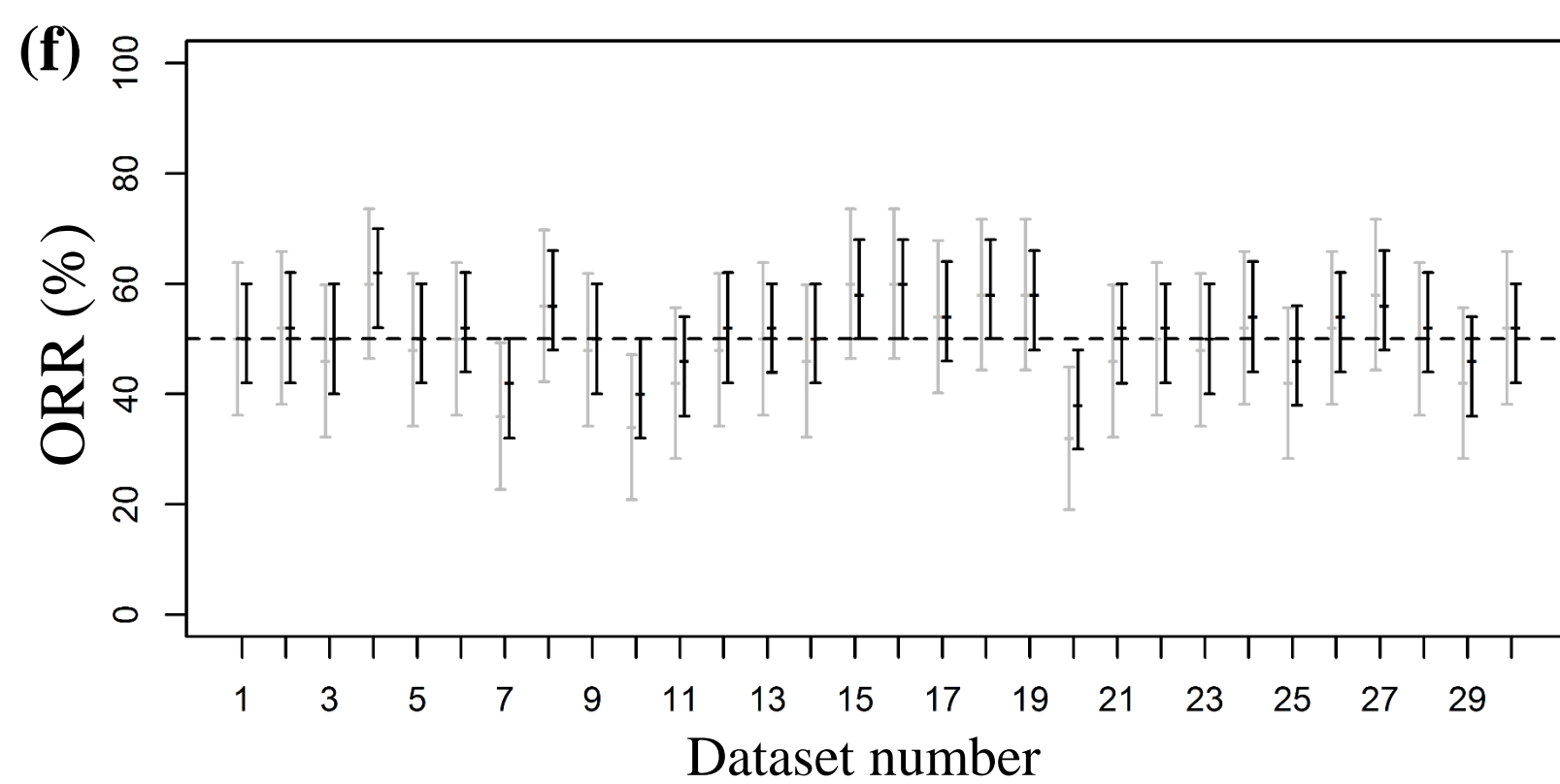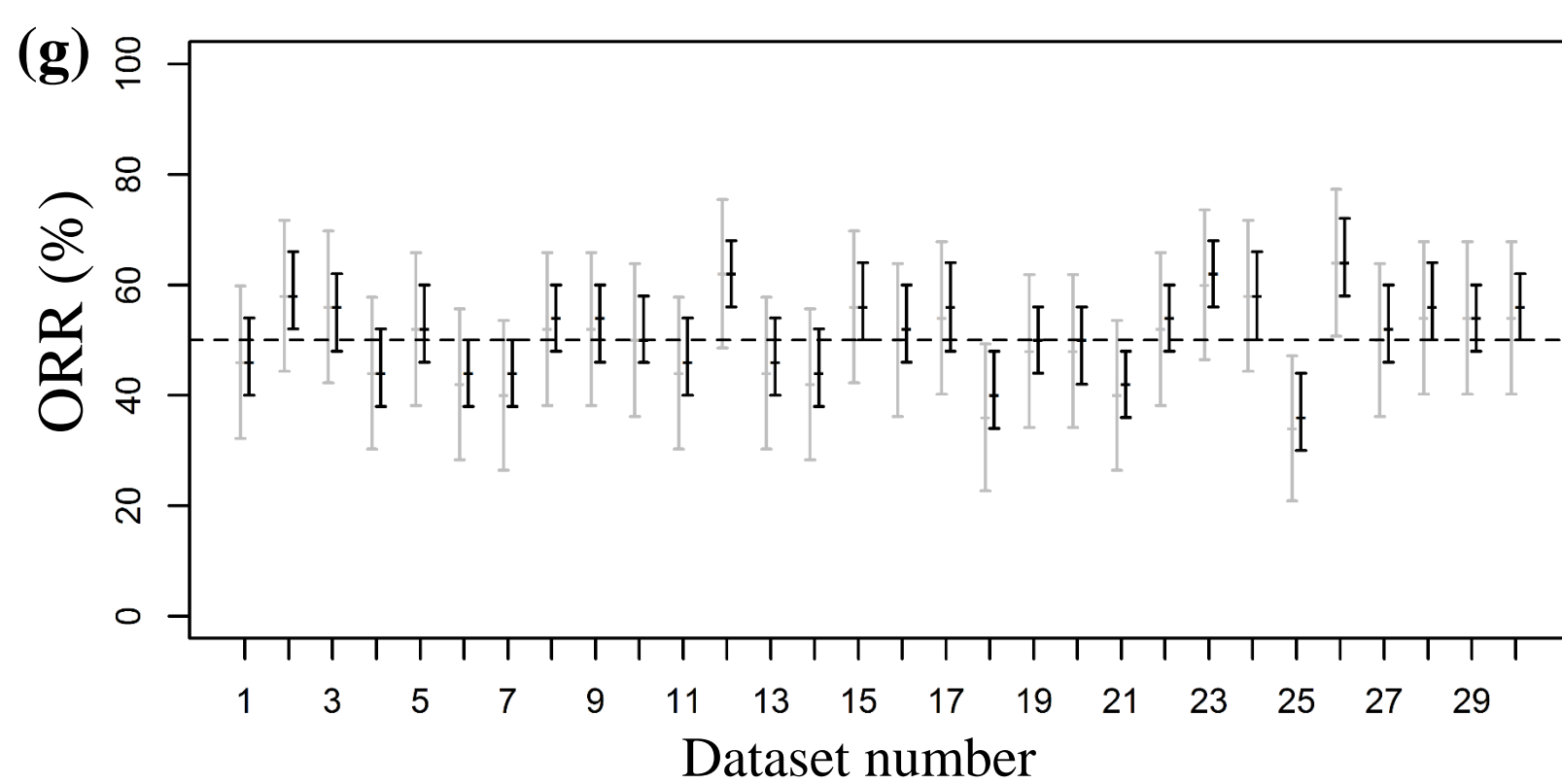

Supplement: Supplementary file 3 — Figure S2. Observed objective response rates (ORR) with 95% confidence intervals and 95% central ranges from the evaluation tool depending characteristics of simulated data sets in which the true ORR is 50%. (a) when the assumed distributions of baseline tumor burden size and percent change are LN(3.55, 0.532) and − 30±N(0, 52), respectively. (b) when the assumed distributions of baseline tumor burden size and percent change are LN(3.55, 1.222) and − 30±N(0, 52), respectively. (c) when the assumed distributions of baseline tumor burden size and percent change are LN(3.55, 0.532) and − 30±N(0, 202), respectively. (d) when the assumed distributions of baseline tumor burden size and percent change are LN(3.55, 1.222) and − 30±N(0, 202), respectively. (e) when the assumed distributions of baseline tumor burden size and percent change are LN(4.25, 0.532) and − 30±N(0, 52), respectively. (f) when the assumed distributions of baseline tumor burden size and percent change are LN(3.55, 0.532) and − 30±N(20, 52), respectively. (g) when the assumed distributions of baseline tumor burden size and percent change are LN(4.25, 0.532) and − 30±N(20, 52), respectively. Dashed line: true ORR; Gray lines: the observed ORR and 95% confidence interval; Black lines: median and 95% central range from the tool. (PDF 323 kb) [file 12874_2019_727_MOESM3_ESM.pdf]

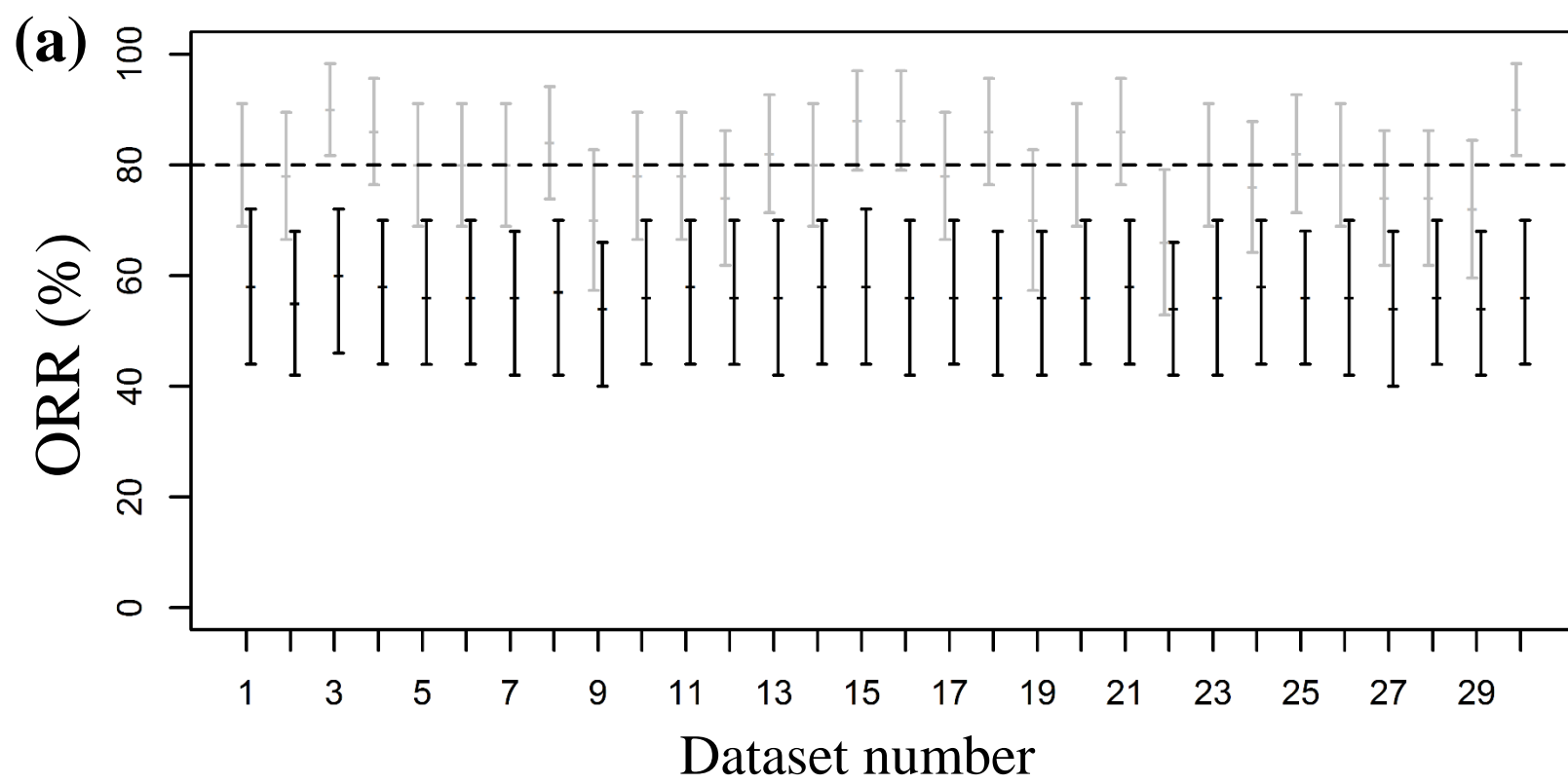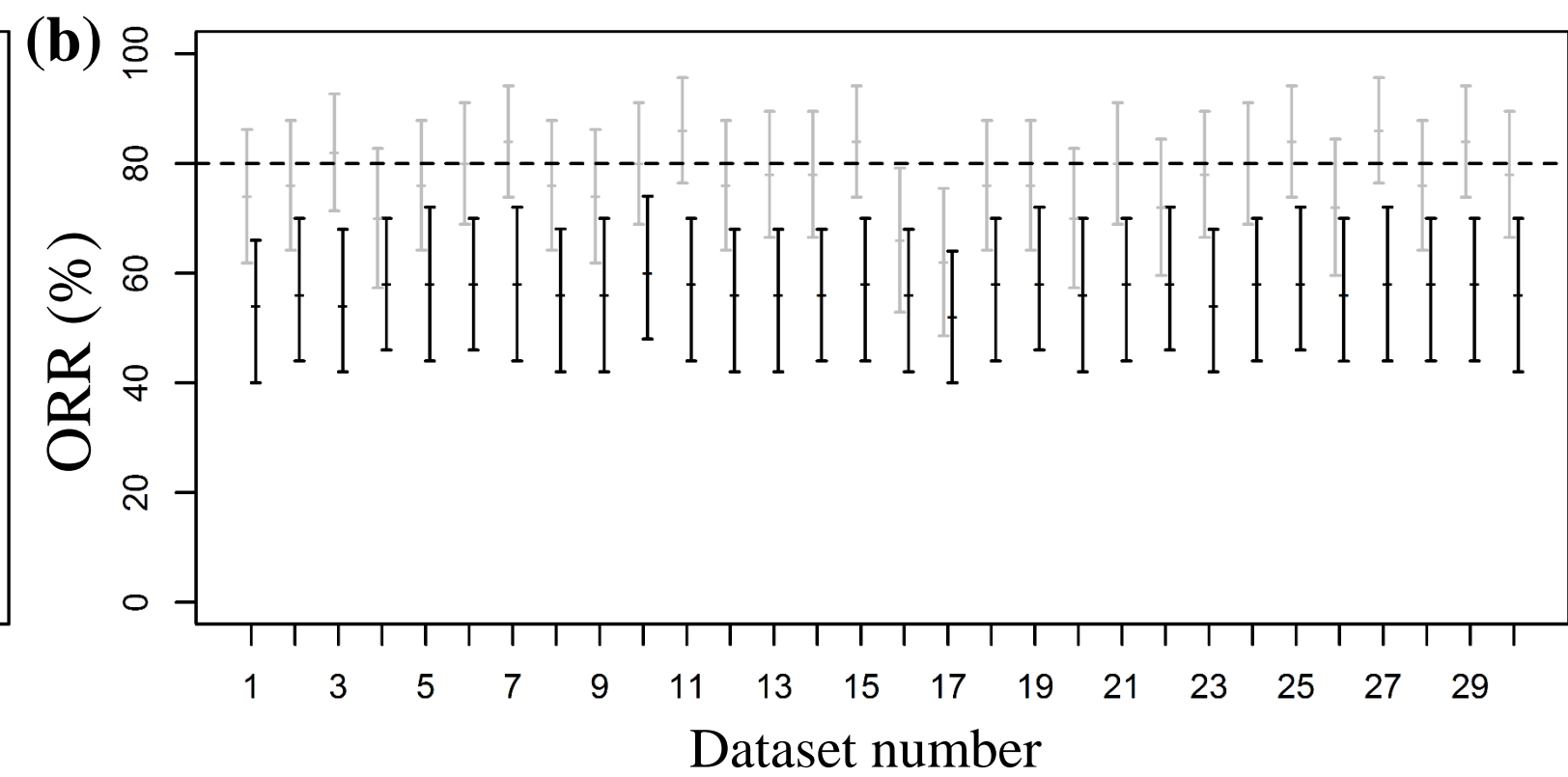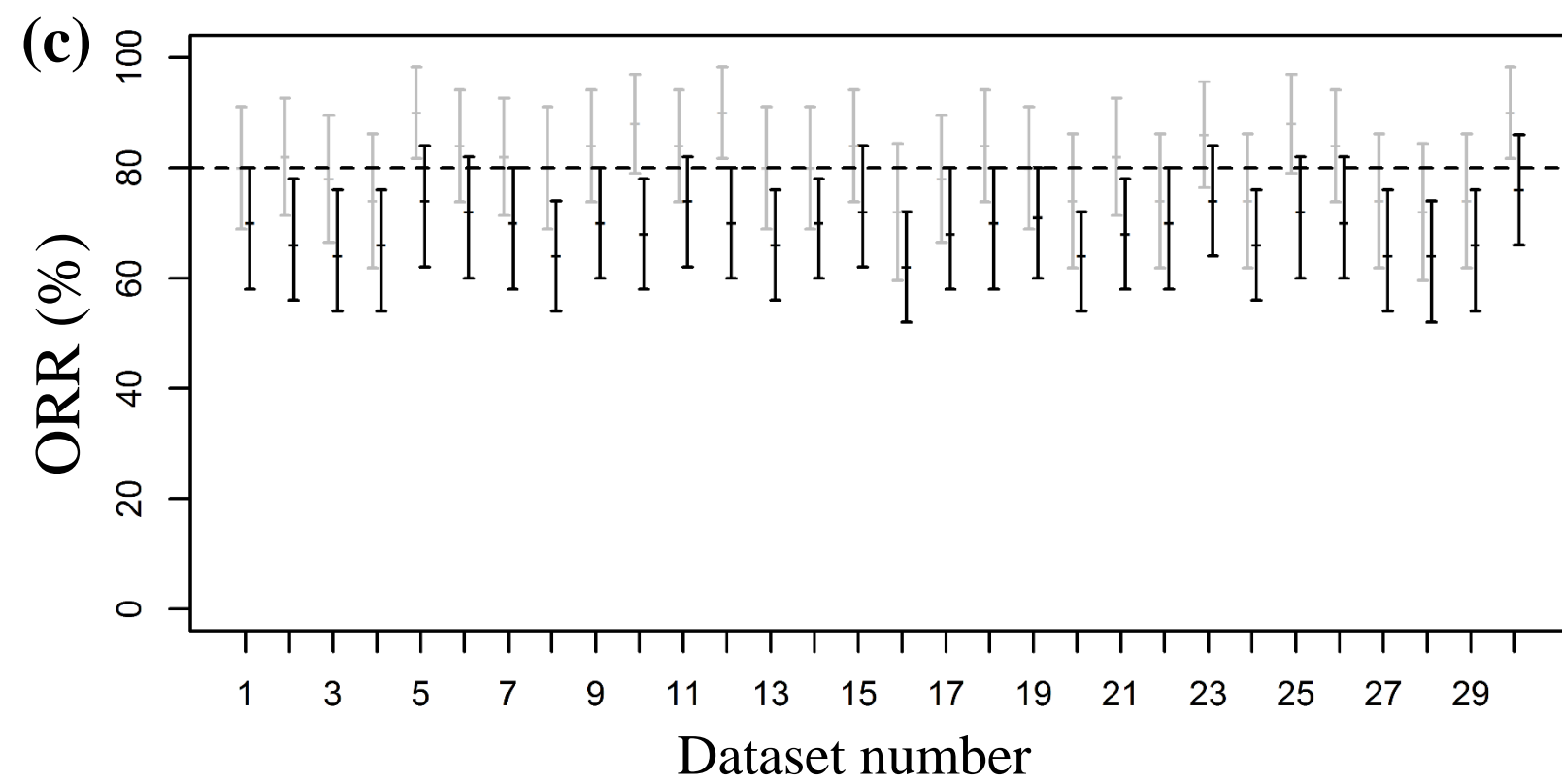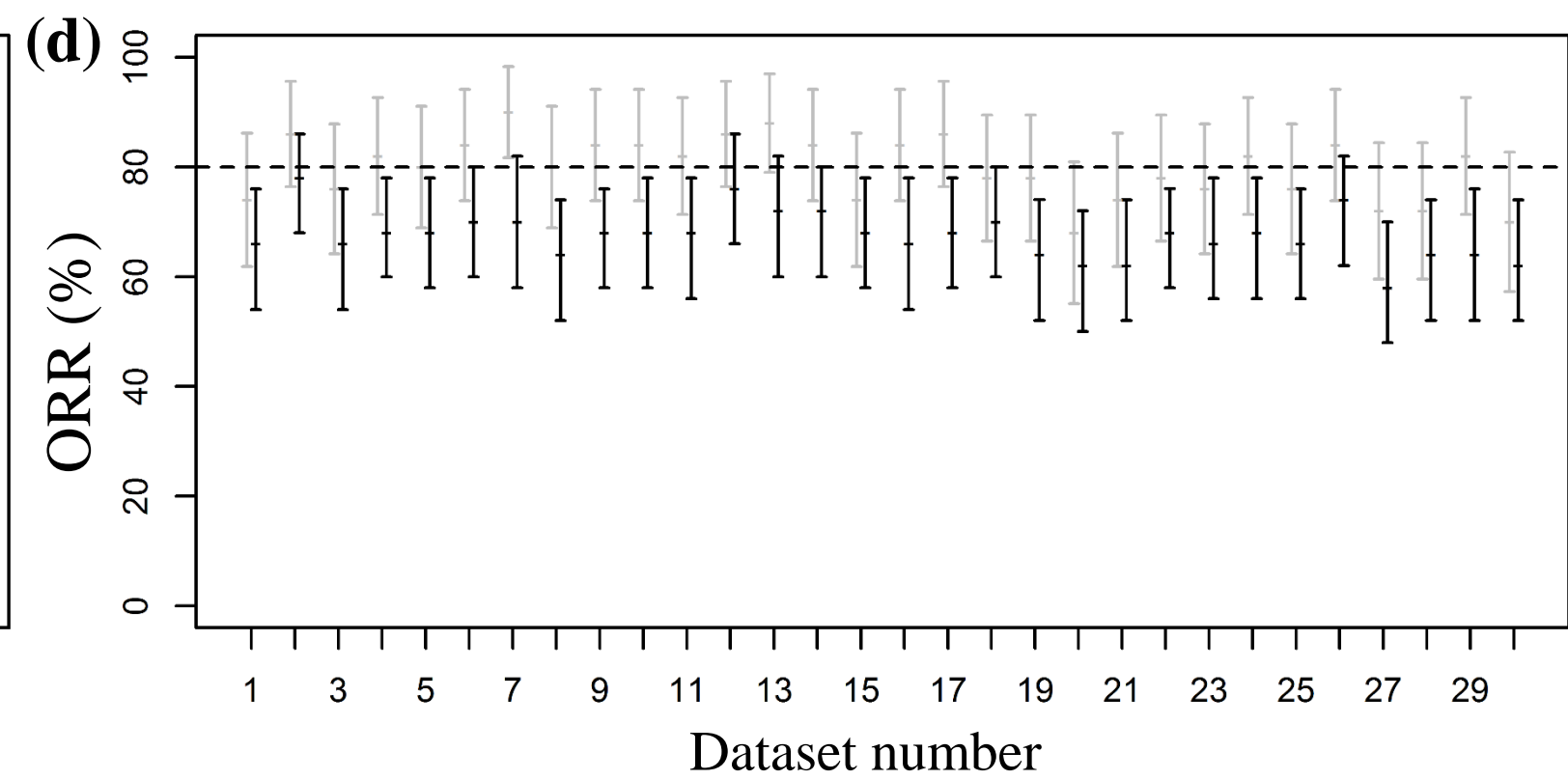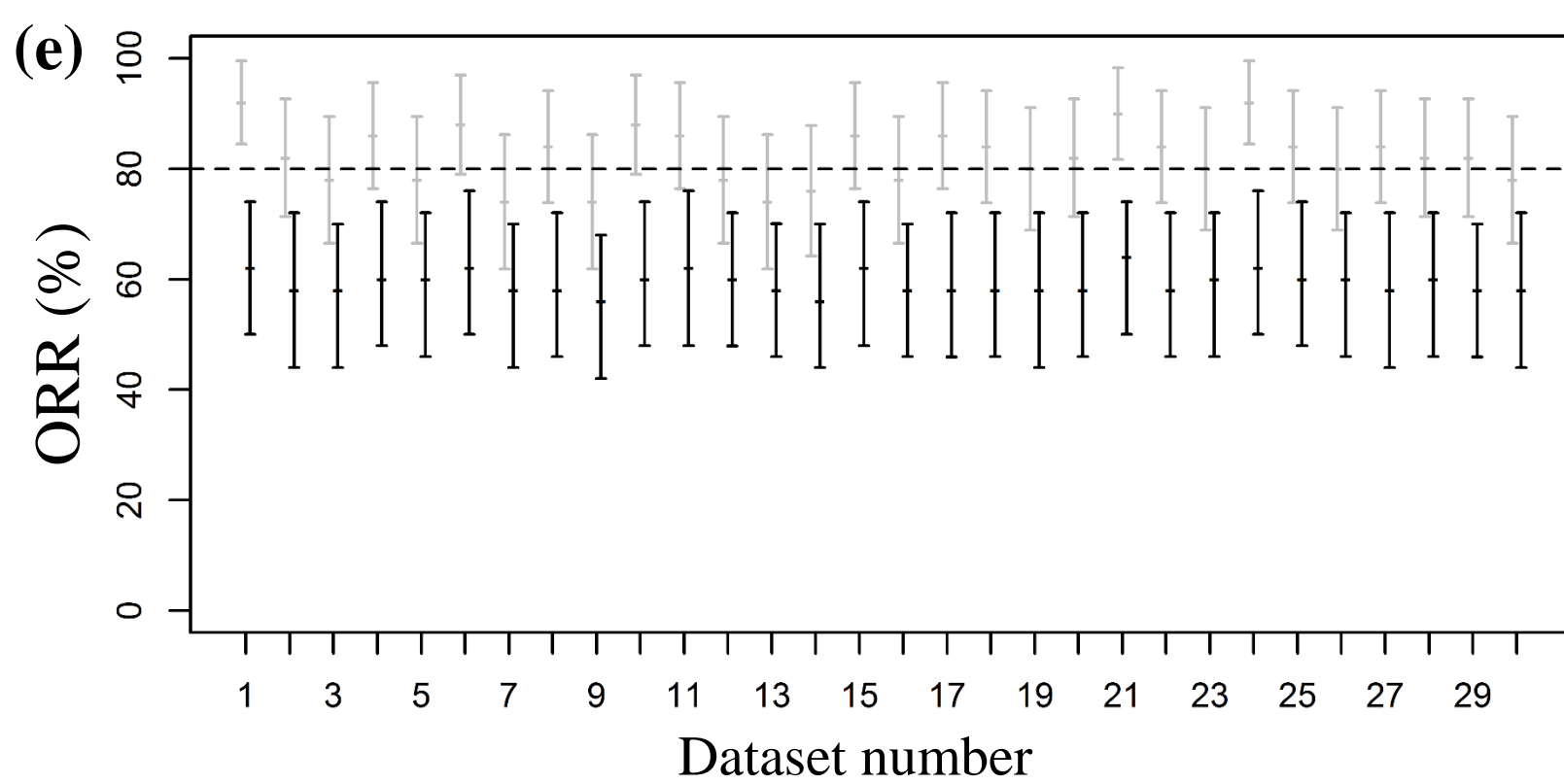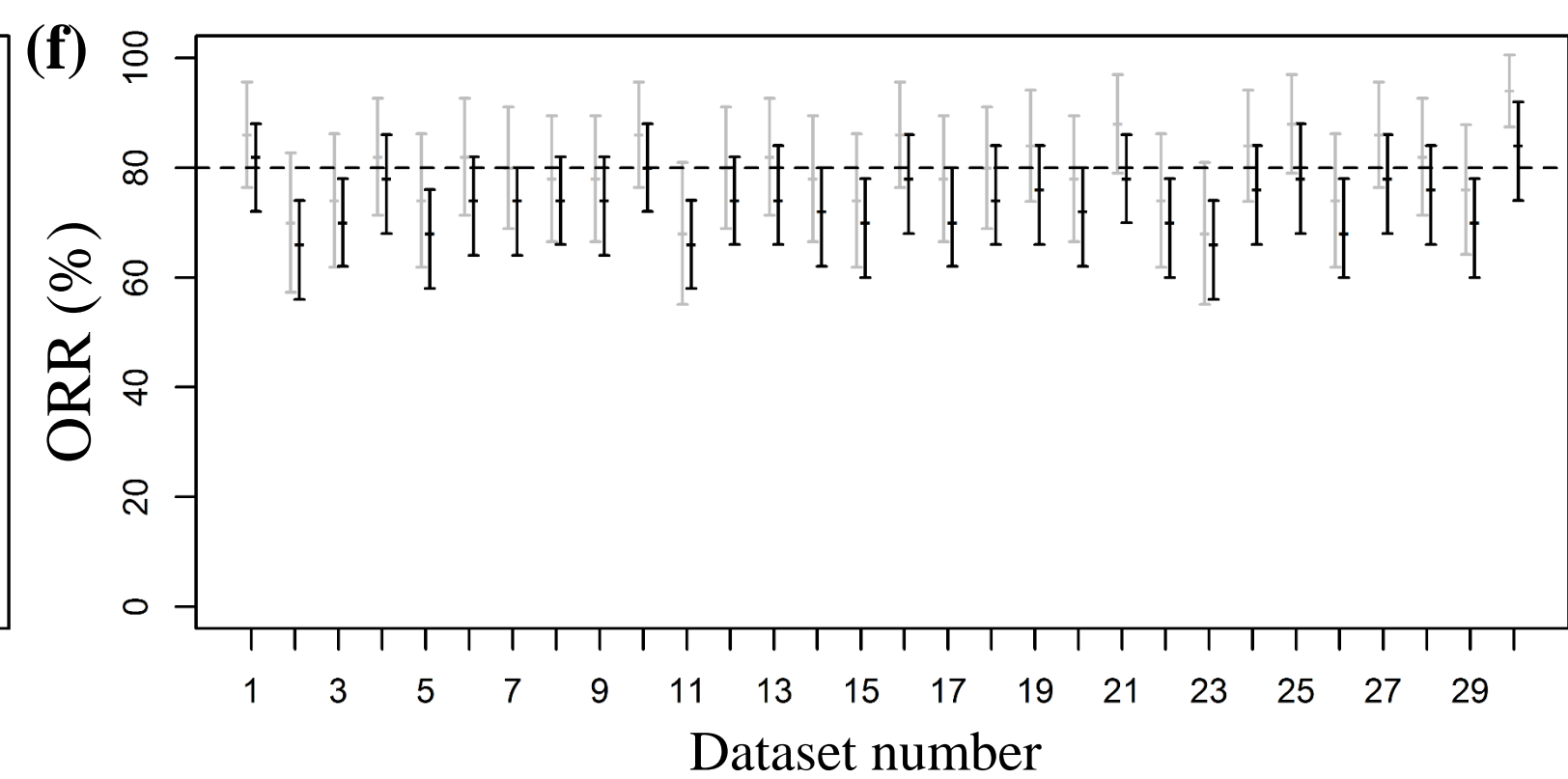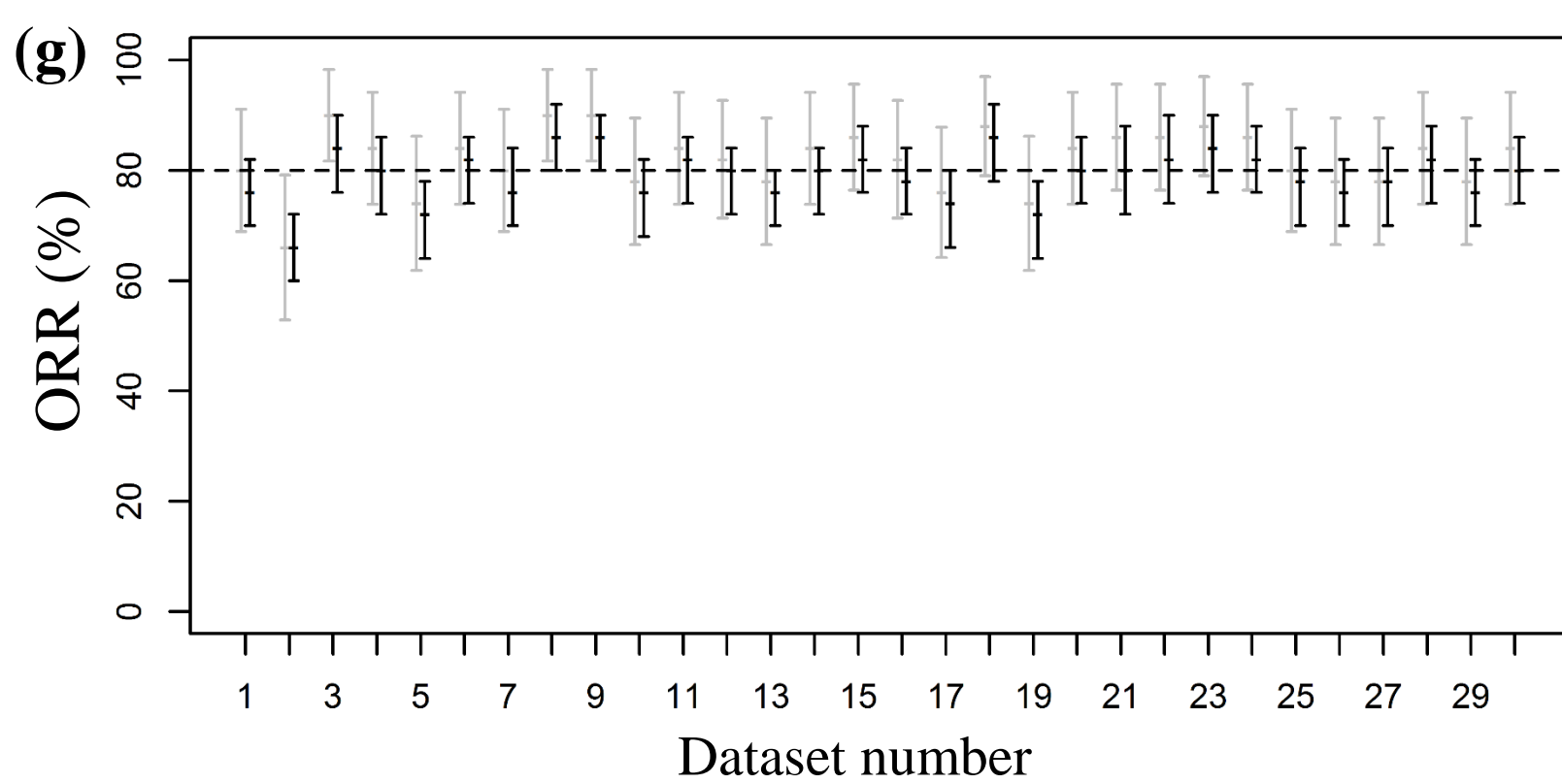

Supplement: Supplementary file 4 — Figure S3. Observed objective response rates (ORR) with 95% confidence intervals and 95% central ranges from the evaluation tool depending characteristics of simulated data sets in which the true ORR is 80%. (a) when the assumed distributions of baseline tumor burden size and percent change are LN(3.55, 0.532) and − 30±N(0, 52), respectively. (b) when the assumed distributions of baseline tumor burden size and percent change are LN(3.55, 1.222) and − 30±N(0, 52), respectively. (c) when the assumed distributions of baseline tumor burden size and percent change are LN(3.55, 0.532) and − 30±N(0, 202), respectively. (d) when the assumed distributions of baseline tumor burden size and percent change are LN(3.55, 1.222) and − 30±N(0, 202), respectively. (e) when the assumed distributions of baseline tumor burden size and percent change are LN(4.25, 0.532) and − 30±N(0, 52), respectively. (f) when the assumed distributions of baseline tumor burden size and percent change are LN(3.55, 0.532) and − 30±N(20, 52), respectively. (g) when the assumed distributions of baseline tumor burden size and percent change are LN(4.25, 0.532) and − 30±N(20, 52), respectively. Dashed line: true ORR; Gray lines: the observed ORR and 95% confidence interval; Black lines: median and 95% central range from the tool. (PDF 329 kb) [file 12874_2019_727_MOESM4_ESM.pdf]
